# Evaluation of direct restorations using the revised FDI criteria: results from a reliability study

**DOI:** 10.1007/s00784-022-04771-9

**Published:** 2022-11-18

**Authors:** Sabine Mesinger, Katrin Heck, Alexander Crispin, Roland Frankenberger, Milena Cadenaro, John Burgess, Arnd Peschke, Siegward D. Heintze, Bas Loomans, Niek Opdam, Reinhard Hickel, Jan Kühnisch

**Affiliations:** 1 Department of Conservative Dentistry and Periodontology, University Hospital, Ludwig Maximilians-University of Munich, Goethestraße 70, 80336 Munich, Germany; 2 Institute of Medical Biometry and Epidemiology, Ludwig-Maximilians-University of Munich, Munich, Germany; 3 Department of Operative Dentistry, Endodontics, and Pediatric Dentistry Medical Center for Dentistry, University Medical Center Giessen and Marburg, Campus Marburg, Marburg, Germany; 4 Department of Medical Sciences, Institute for Maternal and Child Health IRCCS “Burlo Garofolo”, University of Trieste, Via Dell’Istria, Trieste, Italy; 5 School of Dentistry, University of Alabama at Birmingham, Birmingham, AL United States; 6 Research & Development, Ivoclar Vivadent AG, Schaan, Liechtenstein; 7 Department of Dentistry, Radboud Institute for Health Sciences, Radboud University Medical Center, Nijmegen, The Netherlands

**Keywords:** Reliability, Reproducibility, Dental restoration, Diagnostics

## Abstract

**Objectives:**

The purpose of this in vitro reliability study was to determine the intra- and inter-examiner agreement of the revised FDI criteria including the categories “fracture of material and retention” (F1) and “caries at restoration margin” (B1).

**Materials and methods:**

Forty-nine photographs of direct tooth-coloured posterior (*n* = 25) and anterior (*n* = 24) restorations with common deficiencies were included. Ten dental experts repeated the assessment in three blinded rounds. Later, the experts re-evaluated together all photographs and agreed on a reference standard. Statistical analysis included the calculation of Cohen’s (Cκ), Fleiss’ (Fκ), and weighted Kappa (wκ), the development of a logistic regression with a backward elimination model and Bland/Altman plots.

**Results:**

Intra- and inter-examiner reliability exhibited mostly moderate to substantial Cκ, Fκ, and wκ values for posterior restorations (e.g. Intra: F1 Cκ = 0.57, wκ = 0.74; B1 Cκ = 0.57, wκ = 0.73/Inter F1 Fκ = 0.32, wκ = 0.53; B1 Fκ = 0.41, wκ = 0.64) and anterior restorations (e.g. Intra F1 Cκ = 0.63, wκ = 0.76; B1 Cκ = 0.48, wκ = 0.68/Inter F1 Fκ = 0.42, wκ = 0.57; B1 Fκ = 0.40, wκ = 0.51). Logistic regression analyses revealed significant differences between the evaluation rounds, examiners, categories, and tooth type. Both the intra- and inter-examiner reliability increased along with the evaluation rounds. The overall agreement was higher for anterior restorations compared to posterior restorations.

**Conclusions:**

The overall reliability of the revised FDI criteria set was found to be moderate to substantial.

**Clinical relevance:**

If properly trained, the revised FDI criteria set are a valid tool to evaluate direct and indirect restorations in a standardized way. However, training and calibration are needed to ensure reliable application.

**Supplementary Information:**

The online version contains supplementary material available at 10.1007/s00784-022-04771-9.

## Introduction

Adequate dental decision-making requires valid and reliable diagnostic detection, classification and/or assessment systems to evaluate anatomical, physiological, and pathological conditions of the stomatognathic system which includes teeth, periodontium, oral mucosa, alveolar bone, or the temporo-mandibular-joint. For restored teeth, different evaluation systems have been published in the past. Cvar and Ryge [1] published the first evaluation system in 1971. As Dr. Gunnar Ryge, a dentist, was Director of the Materials and Technology Branch of the United States Public Health Service, the guidelines had been called USPHS Criteria [2]. J. Cvar was statistician at USPHS and developed statistical methods to analyse the data. In 1980 Ryge published “modified Ryge/ USPHS Criteria” [3]. An elementary aspect of the USPHS Criteria was the development of criteria for clinical testing and estimation of their reliability [1]. These criteria were well accepted and, are still, used in clinical studies evaluating dental restorations [4]. With the development of dental restorative materials a more discriminative and sensitive scale was needed [5, 6]. In 2007, new clinical criteria for the evaluation of dental restorations were suggested by Hickel et al. and approved by the science Committee of the FDI World Dental Federation [5, 6], which are nowadays commonly known as “FDI criteria.” In 2010, some modifications to the original criteria set and clinical examples have been published by the same workgroup [7, 8]. In brief, the diagnostic system classifies aesthetic, functional, and biological properties and covers various types of failures with five grades for each criterion. In detail, scores 1 to 3 indicated clinically acceptable restorations, and scores 4 and 5 summarized clinically unacceptable situations indicating repair (score 4) or replacement (score 5). In 2019, a workgroup started to update the FDI criteria for the clinical evaluation of dental restorations by using a stepwise, consensus-based process to improve the clinical usability, practicability and acceptability. Aiming at excluding subjectivity and supporting data-based decision-making, it was suggested to prove the diagnostic reliability at important project milestones. Beside this need, the reliability of the FDI criteria has been scarcely addressed so far and conflicting data are documented in the literature. Perdigao et al. [9] reported on an excellent inter-examiner reliability, whereas Kim et al. [10] documented inconsistent results concerning the intra- and inter-examiner reliability when applying the FDI criteria in direct tooth-coloured posterior restorations using intraoral digital photographs. In addition, the authors referred to the subjectivity of the criteria set which may hinder a reproducible decision making [10] and support the need for an update.

Taking into account the previously mentioned facts, it became evident that it is reasonable to conduct a reliability study parallel to the revision of the criteria set and to provide the data. Therefore, the aim of this in vitro reliability study was to evaluate the intra- and inter-examiner reliability during the revision of the FDI criteria exemplary for direct tooth-coloured anterior and posterior restorations by use of intraoral photographs.

## Methods and materials

This in vitro diagnostic study was approved by the local Ethics Committee (Project No. 19–185). The reporting of this investigation followed the Guidelines for Reporting Reliability and Agreement Studies (GRRAS) [11].

### Expert group

A group of 10 dentists from Europe and North America participated as experts in the update process of the criteria set. The expert group represented a broad spectrum of clinical and scientific experience in the field of restorative dentistry, and each of the expert contributed to the revised FDI criteria set. Details of the update process were reported elsewhere [12]. It is noteworthy that all participating experts were familiar with the concept of the FDI criteria and no specific theoretical or practical training was performed before each round of evaluation.

### Set of intraoral photographs

For this investigation, a few thousand anonymised intraoral photographs not older than 10 years from case documentations or earlier clinical studies conducted at the Department of Conservative Dentistry and Periodontology were screened for the presence of typical failures on direct tooth-coloured restorations. Photographs of direct restorations made of amalgam, temporary filling materials, and all types of indirect restorations were not considered. In a next step, S. Mesinger (SM), J. Kühnisch (JK), and R. Hickel (RH) identified ~ 100 photographs according to the following inclusion criteria: (1) one direct tooth-coloured anterior (labial aspect) or posterior restoration (occlusal aspect) made of composite, compomer, or glass ionomer cement in the centre of the image; (2) broad spectrum of failures, e.g. different stages of material fracture to the extent of complete loss of retention; (3) photograph with good contrast, brightness and sharpness. Finally, the Munich group has chosen 49 photographs with a well-balanced distribution of posterior (*n* = 25) and anterior (*n* = 24) teeth and assigned a unique identification number to each image. Furthermore, a mark-up was embedded on each image to highlight the relevant restoration to avoid miss-classifications in case of multiple fillings per tooth.

### Expert evaluations and stepwise revision

The evaluation of the intraoral photographs was performed using an online survey platform (www.SoSciSurvey.com, SoSci Survey GmbH, Munich, Germany). An individual, blinded, and independent access was provided for each participating expert (*n* = 10) and evaluation round (*n* = 3). All photographs were evaluated by all experts (fully crossed design), according to the most recent version of the revised criteria set. To decrease recognition and recall of the photographs during the study period, the sequence of images was randomly changed between the first, second and third round of evaluations. All evaluation rounds were performed after alterations of the criteria set were made on the basis of the current literature, clinical and scientific experiences, and the ongoing discussions in the expert group. After each round, the feedback from all experts was collected, condensed, and incorporated into an updated criteria version. In addition, the results from the statistical analyses of the intra- and inter-examiner reliability were compiled and discussed during online meetings. This led to modifications in the criteria set with the aim to improve the precision of each criterion. During the first two rounds, all experts scored each image according to the 5-point scale of each criterion which resulted into an *ordinal* data set. Importantly, after the second evaluation round, it became obvious that some scores make the evaluation in other categories irrelevant and, therefore, the score “not applicable” was integrated. Subsequently, all evaluations were repeated in a third round after finalisation of the revision process and all experts scored each photograph according to the 5-point scale plus the “not applicable” score which resulted into a *nominal* data set. It is noteworthy to point out that the scoring criteria changed between the three evaluation rounds.

The selected criteria suitable for the evaluation on intraoral photographs were the following: F1 — fracture of material and retention, F2 — marginal adaptation, F4 — form and contour, B1 — caries at restoration margin (CAR), B2 — dental hard tissue defects, A1 — surface lustre and texture, A2 — marginal staining, and A3 — colour match.

### Consensus decision (reference standard)

After all three evaluation rounds, the expert group re-assessed all intraoral images during two online meetings in December 2020, compared their individual results with those of the others, and determined a consensus decision for each restoration and categories. The 5-point scale plus the “not applicable” score was used again which resulted into a *nominal* data set for the reference standard.

### Data management and statistical analysis

All data of each round of evaluation (*N* = 3), all experts (*N* = 10), and the reference standard were collected on an online survey platform (www.SoSciSurvey.com, SoSci Survey GmbH, Munich, Germany). Later, the data was exported into an Excel spreadsheet (Excel 2016, Microsoft, Redmond, WA, USA) and checked for plausibility before analysis. The descriptive and explorative data analysis was performed using Excel and SPSS (SPSS Statistics 27, 2020, IBM corporation, Armonk, NY, USA). With respect to the described process ordinal-scaled data from the 1st and 2nd round of evaluation and nominal-scaled data from the evaluations 3rd round of evaluation and reference standard were analysed. However, this aspect resulted in the need of different statistical methods to handle the data.

The analysis was computed for each criterion and each expert, in relation to posterior and anterior restorations (tooth type) as well as the three evaluation rounds. The descriptive analysis included the calculation of the percentage of agreement for the intra- and inter-examiner reliability among the experts and in relation to the reference standard. For the explorative analysis of the *nominal* data set, Cohen´s Kappa (Cκ) was computed for the intra-examiner reliability and Fleiss´ Kappa (Fκ) for the inter-examiner reliability. Additionally, for the explorative analysis of the observation as *ordinal* data set, linear weighted Kappa (wκ) estimates were computed for the intra-examiner reliability. For the inter-examiner reliability, linear weighted Kappa (wκ) was calculated for all coder pairs using SPSS. To provide an overall value, the arithmetic mean of these estimates was calculated with Excel [13, 14]. The same procedure was applied for the reliability of all examiners in relation to the reference standard. Kappa values within the below-mentioned ranges need to be interpreted as follows: 0.0 to 0.2 — slight agreement, 0.21 to 0.40 — fair agreement, 0.41 to 0.60 — moderate agreement, 0.61 to 0.80 — substantial agreement, and 0.81 to 1.00 — (almost) perfect agreement [15]. Furthermore, modified Bland/Altman plots [16, 17] and binominal logistic regression analysis using a backward elimination model were performed and used for exploring all diagnostic decisions in relation to the reference standard. The analysis was computed with the data from all rounds of evaluation, examiners, categories and tooth type (anterior/posterior).

## Results

Tables 1, 2 and 3 give an overview of all percentage agreements and Kappa values of the intra- and inter-examiner reliability in relation to the chosen FDI criteria. The *intra*-examiner reliability was mainly documented as substantial for all criteria in posterior teeth with the highest Kappa values for “colour match/A3” (Cκ 0.71, wκ 0.76), “marginal adaptation/ F2” (Cκ 0.66, wκ 0.75), “fracture of material and retention/F1” (Cκ 0.57, wκ 0.74), and “caries at restoration margin/B1” (Cκ 0.57, wκ 0.73). In anterior restorations, the highest Kappa values were computed for “fracture of material and retention/F1” (Cκ 0.63, wκ 0.76), “marginal adaptation/F2” (Cκ 0.48, wκ 0.61), “caries at restoration margin/B1” (Cκ 0.48, wκ 0.68), and again “marginal staining/A2” (Cκ 0.55, wκ 0.67). The *inter*-examiner reliability was mostly in the moderate range (fair to substantial for posterior restorations and slight to moderate in anterior restorations). For posterior restorations, the highest Kappa values were documented for the criteria “caries at restoration margin/B1” (Fκ 0.41, wκ 0.64), “form and contour/F4” (Fκ 0.46, wκ 0.49), “fracture of material and retention/F1” (Fκ 0.32, wκ 0.53), and “marginal adaptation/F2” (Fκ 0.34, wκ 0.52). In anterior restorations, the highest weighted Kappa values were reached for the criterion “marginal staining/A2” (Fκ 0.41, wκ 0.56), and also “fracture of material and retention/F1” (Fκ 0.42, wκ 0.57), and “caries at restoration margin/B1” (Fκ 0.40, wκ 0.51).

**Table 1:** Inter-examiner reliability values for direct tooth-coloured posterior restorations across 10 examiners and in relation to the selected FDI criteria

Posterior restorations		Inter-examiner reliability				Reliability in relation to reference standard	
		Nominal data set (scores 1–5 incl. “not applicable”)		Ordinal data set (scores 1–5 only)		Ordinal data set (scores 1–5 only)	
Criteria	Evaluation round	% Agreement	Fleiss Kappa	% Agreement	Weighted Kappa	% Agreement	Weighted Kappa
F1	1	42.2	0.28	44.4	0.48	53,9	0.59
	2	45.7	0.33	50.5	0.54	63,3	0.66
	3	45.2	0.32	49.2	0.53	61.8	0.68
F2	1	48.3	0.36	41.5	0.54	51,6	0.60
	2	45.7	0.33	38.7	0.50	51,0	0.59
	3	46.7	0.34	41.9	0.52	53.3	0.58
F4	1	50.4	0.39	40.2	0.38	51,1	0.47
	2	54.9	0.43	45.3	0.41	56,7	0.53
	3	57.8	0.46	49.0	0.49	60.0	0.59
B1	1	48.2	0.29	50.1	0.54	61,5	0.65
	2	53.3	0.35	55.0	0.58	67,0	0.70
	3	59.9	0.41	66.9	0.64	78.8	0.78
B2	1	48.4	0.26	47.4	0.43	56,8	0.55
	2	50.4	0.28	48.3	0.43	59,6	0.55
	3	57.8	0.34	62.9	0.49	64.2	0.59
A1	1	46.4	0.29	39.8	0.28	56,8	0.44
	2	47.6	0.31	44.8	0.31	58,4	0.48
	3	52.4	0.37	48.3	0.41	65.6	0.58
A2	1	52.0	0.38	41.2	0.37	49,5	0.38
	2	53.1	0.39	42.9	0.32	54,2	0.46
	3	51.8	0.38	40.0	0.34	53.9	0.49
A3	1	55.4	0.34	62.2	0.45	77,0	0.61
	2	56.9	0.37	62.2	0.48	78,0	0.66
	3	51.8	0.28	58.8	0.40	74.4	0.62

Criteria: F1: fracture of material and Retention, F2: marginal adaptation, F4: form and contour, B1: caries at restoration margin (CAR), B2: dental hard tissue defects, A1: surface lustre and texture, A2: marginal staining, A3: colour match

**Table 2:** Inter-examiner reliability values for direct tooth-coloured anterior restorations across 10 examiners and in relation to the selected FDI criteria

Anterior restorations		Inter-examiner reliability				Reliability in relation to reference standard	
		Nominal data set (scores 1–5 incl. “not applicable”)		Ordinal data set (scores 1–5 only)		Ordinal data set (scores 1–5 only)	
Criteria	Evaluation round	% Agreement	Fleiss Kappa	% Agreement	Weighted Kappa	% Agreement	Weighted Kappa
F1	1	53.5	0.34	51.5	0.54	60.5	0.52
	2	62.1	0.41	60.5	0.58	65.2	0.59
	3	61.9	0.42	60.3	0.57	64.8	0.61
F2	1	36.0	0.18	33.8	0.25	44.8	0.39
	2	37.8	0.20	35.6	0.34	45.2	0.46
	3	43.2	0.27	42.0	0.44	51.0	0.55
F4	1	46.7	0.33	36.0	0.34	48.5	0.45
	2	54.8	0.42	45.8	0.40	62.0	0.55
	3	54.9	0.40	45.9	0.33	60.5	0.55
B1	1	62.0	0.36	60.2	0.43	71.4	0.58
	2	65.3	0.42	65.8	0.51	72.6	0.63
	3	65.7	0.40	60.4	0.51	71.8	0.66
B2	1	59.9	0.32	57.5	0.19	68.6	0.34
	2	60.6	0.32	59.0	0.26	72.9	0.43
	3	69.0	0.38	69.1	0.25	77.5	0.47
A1	1	44.3	0.24	39.2	0.26	45.0	0.32
	2	50.6	0.32	46.3	0.38	49.0	0.37
	3	54.4	0.35	50.8	0.35	65.5	0.54
A2	1	53.5	0.39	49.3	0.55	56.8	0.61
	2	52.8	0.37	49.5	0.53	61.0	0.64
	3	55.9	0.41	53.6	0.56	68.5	0.71
A3	1	45.6	0.24	40.7	0.24	54.5	0.34
	2	46.9	0.28	42.4	0.30	56.2	0.40
	3	56.0	0.36	52.0	0.34	66.5	0.50

Criteria: F1: fracture of material and Retention, F2: marginal adaptation, F4: form and contour, B1: caries at restoration margin (CAR), B2: dental hard tissue defects, A1: surface lustre and texture, A2: marginal staining, A3: colour match

**Table 3:** Intra-examiner reliability values for direct tooth-coloured posterior and anterior restorations across 10 examiners in relation to the selected FDI criteria

Intra-examiner reliability		Posterior restorations				Anterior restorations			
		Nominal data set (scores 1–5 incl. “not applicable”)		Ordinal data set (scores 1–5 only)		Nominal data set (scores 1–5 incl. “not applicable”)		Ordinal data set (scores 1–5 only)	
Criteria	Evaluation round	% Agreement	Cohen Kappa	% Agreement	Weighted Kappa	% Agreement	Cohen Kappa	% Agreement	Weighted Kappa
F1	1 vs. 2	66.4	0.57	67.5	0.74	74.2	0.63	73.3	0.76
	2 vs. 3	58.4	0.48	59.7	0.66	70.0	0.54	69.3	0.68
F2	1 vs. 2	73.2	0.66	72.8	0.75	62.5	0.49	62.2	0.60
	2 vs. 3	67.2	0.59	67.2	0.72	61.7	0.48	62.0	0.61
F4	1 vs. 2	72.8	0.65	67.5	0.67	69.2	0.60	63.0	0.60
	2 vs. 3	70.0	0.61	64.3	0.62	66.7	0.56	60.0	0.56
B1	1 vs. 2	62.4	0.47	63.3	0.66	72.1	0.49	71.3	0.63
	2 vs. 3	70.4	0.57	72.5	0.73	73.3	0.48	72.8	0.68
B2	1 vs. 2	69.2	0.51	68.6	0.65	75	0.54	74.9	0.51
	2 vs. 3	68.4	0.51	68.9	0.64	75.8	0.57	75.8	0.47
A1	1 vs. 2	71.6	0.61	68.4	0.63	59.6	0.44	56.1	0.47
	2 vs. 3	66.8	0.56	62.7	0.59	67.5	0.54	65.3	0.59
A2	1 vs. 2	70.8	0.61	64.2	0.60	68.3	0.57	66.1	0.57
	2 vs. 3	70.8	0.61	64.9	0.61	56.8	0.55	64.2	0.67
A3	1 vs. 2	80.8	0.71	80.2	0.76	63.3	0.47	60.3	0.50
	2 vs. 3	76.1	0.64	77.4	0.70	67.9	0.54	65.4	0.58

Criteria: F1: fracture of material and Retention, F2: marginal adaptation, F4: form and contour, B1: caries at restoration margin (CAR), B2: dental hard tissue defects, A1: surface lustre and texture, A2: marginal staining, A3: colour match

**Fig. 1:**
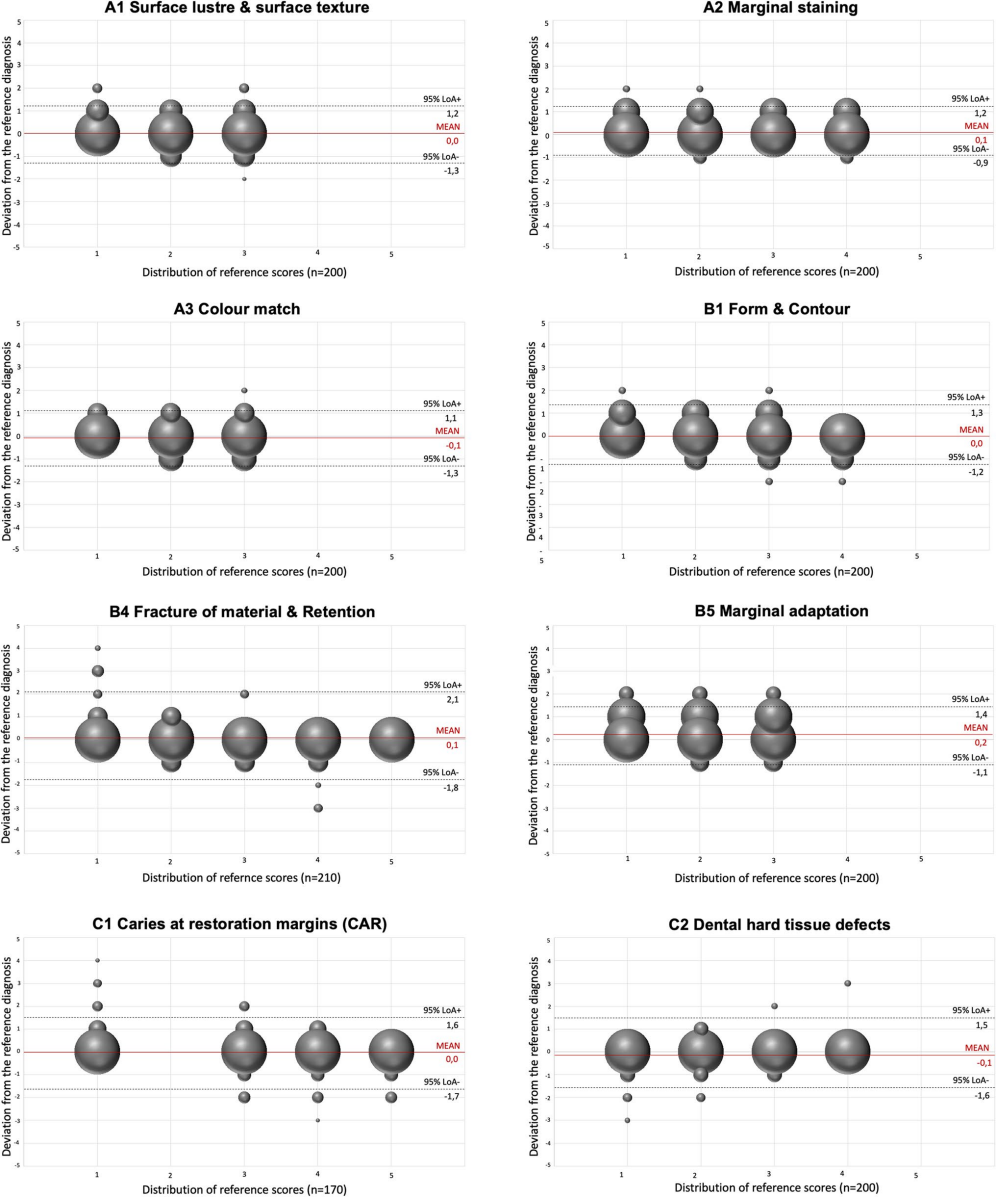
The modified Bland/Altman plots illustrate the agreement in relation to the reference standard for all examiners in the third evaluation round for *posterior teeth*. The size of the bubble correlates with the number of decisions. Ideally, all decision should be located on the Z-line and indicate a perfect agreement

**Fig. 2:**
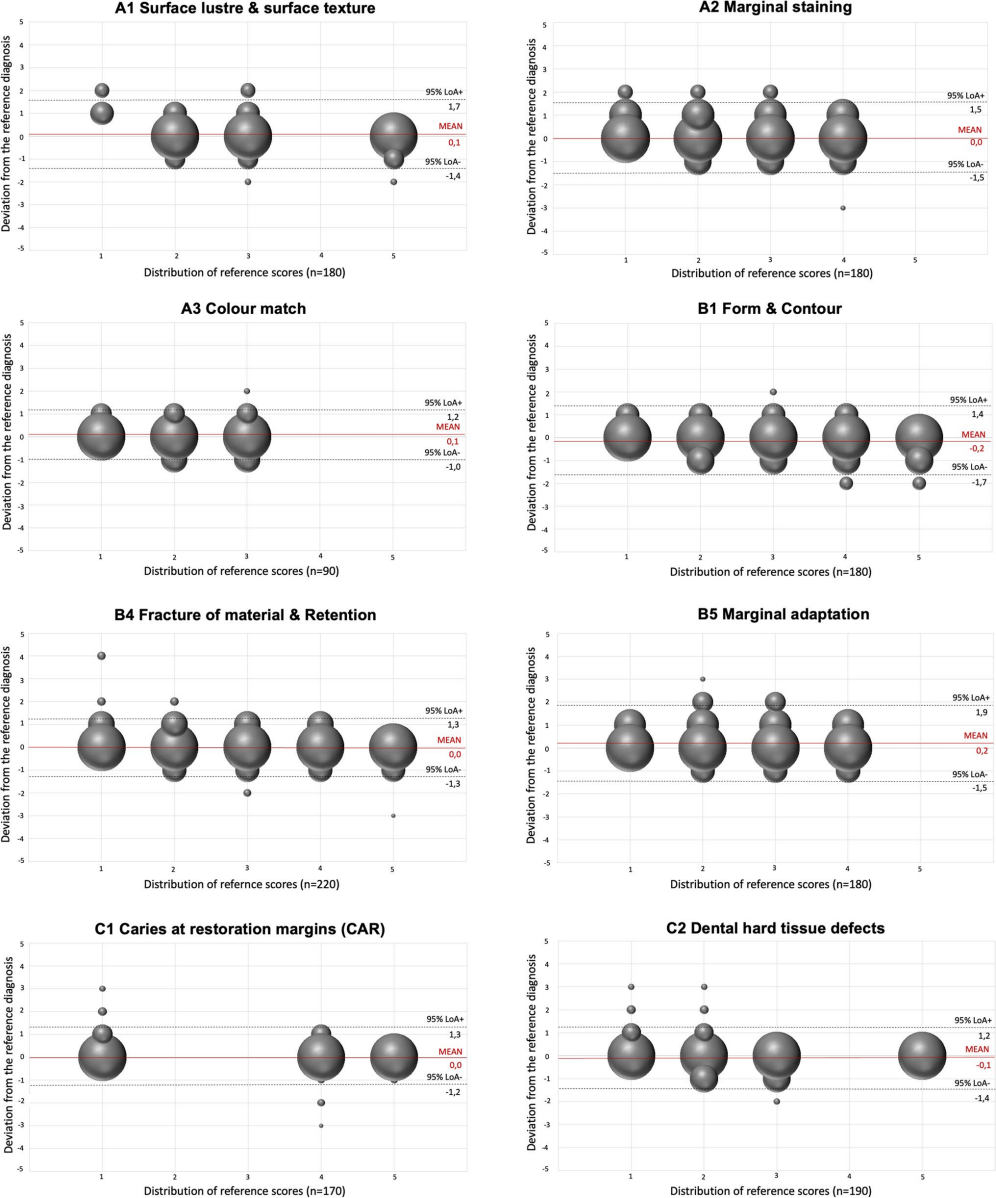
The modified Bland/Altman plots illustrating the agreement against the reference standard for all examiners in the third evaluation round for *anterior teeth*. The size of the bubble correlates with the number of decisions. Ideally, all decision should be located on the Z-line and indicate a perfect agreement

The level of agreement in comparison to the reference standard increased significantly over the three evaluation rounds (Tables 1, 2, and 3), e.g. “caries at restoration margin/B1” (wκ 0.65, 0.70 vs. 0.78) and “fracture of material and retention/F1” (wκ 0.59, 0.66 vs. 0.68) for posterior restorations, and e.g. “marginal staining/A2” (wκ 0.61, 0.64 vs. 0.71), “caries at restoration margin/B1” (wκ 0.58, 0.63 vs. 0.66), and “fracture of material and retention/F1” (wκ 0.52, 0.59 vs. 0.61) for anterior restorations (Tables 2 and 3). For the third evaluation round, the agreement in relation to the reference standard is illustrated in Figs. 1 and 2; deviations from the reference standard were mostly observed in the range of one score only.

The reliability data were further explored by using binominal logistic regression models. In a first attempt data from all evaluation rounds, examiners, categories, and tooth type (anterior/posterior) were analysed. At this step, significant differences between the evaluation rounds became obvious. In detail, it was shown that the reliability increased steadily with each evaluation round (1st round: adjusted odds ratio (aOR) = 1.0; 2nd round aOR = 1.15 with a 95% confidence interval 1.04–1.27; 3rd round: aOR = 1.43 with a 95% CI 1.29–1.58); the difference between each round was statistically significant: 1st vs 2nd round: 0.005/ 2nd vs 3rd round: < 0.001. Therefore, it was decided to include only data from the third evaluation round in the final binominal logistic regression analysis which are shown in Table 4. When considering the rating ability of the examiners in relation to the reference standard, examiner 5 scored closer to the consensus decision in comparison to others, e.g. examiner 8, 9 and 10. Significant differences were also observed between the categories “caries at restoration margin/B1” and “dental hard tissue defects at restoration margin/B2” which were scored with a higher reliability compared to “marginal adaptation/F2.”

**Table 4:** Adjusted odds ratio (aOR) with the corresponding 95% confidence intervals (CI) and *p*-values were computed according to the binominal logistic regression model using backward elimination in relation to the reference standard for the third evaluation round. aOR values lower/higher than 1 indicate a lower/higher agreement in comparison to the diagnostic reference standard and the chosen reference variable (*). Bold numbers highlight a statistically significant influence

Co-variables	Group	aOR	95% CI	*p*-value
Examiner	1*	1	-	-
	2	0.92	0.66–1.27	0.605
	3	0.73	0.53–1.01	0.057
	4	0.87	0.63–1.21	0.395
	5	**1.59**	**1.12–2.25**	**0.010**
	6	0.77	0.56–1.06	0.107
	7	0.73	0.53–1.00	0.050
	8	**0.60**	**0.43–0.83**	**0.002**
	9	**0.68**	**0.50–0.94**	**0.019**
	10	**0.45**	**0.33–0.62**	** < 0.001**
Category	F1*	1	-	-
	F2	**0.67**	**0.51–0.87**	**0.003**
	F4	0.89	0.68–1.18	0.419
	B1	**1.81**	**1.36–2.42**	** < 0.001**
	B2	**1.34**	**1.01–1.77**	**0.039**
	A1	1.13	0.86–1.49	0.393
	A2	0.95	0.72–1.25	0.695
	A3	1.22	0.91–1.65	0.185
Tooth type	Anterior*	1	-	-
	Posterior	**0.85**	**0.73–0.98**	**0.023**

## Discussion

This reliability study supported the recently initiated revision of the FDI criteria set for the evaluation of direct and indirect dental restorations [12]. The reliability tests were carried out together with the revision of the FDI criteria set. The statistical data of the reliability test contributed to several modifications and corroborated the expert´s consensus. It was shown that (1) the intra- and inter-examiner reliability increased over the three evaluation rounds and ranged from a moderate to substantial order of magnitude and (2) Kappa estimates were found to be higher for the functional and biological categories compared to the aesthetic categories (Tables 1, 2, 3, and 4; Figs. 1 and 2).

The results were mostly better or approximately the same compared to reliability tests that were done earlier [10, 18, 19]. The agreement rate increased significantly over the three evaluation rounds. Along with the whole revision process the reliability test contributed to the improved structuring of the criteria set by reducing ambiguous allocations and scoring. It has to be pointed out that the inclusion of principle instructions for use, additional comments, and the score “not applicable” increased a more straightforward decision, especially for complex clinical situations. This might have been the major reason for the significant improvement of the overall reliability after the second evaluation round.

The reliability varied among examiners, categories, and tooth type (anterior/posterior) (Table 4). The highest weighted Kappa values in relation to the reference standard (Tables 1, 2, and 3 ) were registered for “caries at restoration margin,” “fracture of material and retention,” “marginal adaptation,” and “dental hard tissue defects at restoration margin.” The adjusted odds ratio values indicated that the biological criteria “caries at restoration margin” and “dental hard tissue defects at restoration margins” had the best agreement in relation to the reference standard. A significantly lower agreement rate was found for the criterion “marginal adaptation.” The aesthetic criteria — “surface lustre and texture,” “marginal staining,” and “colour match” — as well as the functional criteria “form and contour” showed only a moderate level of agreement (Table 4) which indicates that the assessment of the aesthetical properties of a restoration is somehow subjective and the individual perception of aesthetics by the examiner influences the scoring [10, 20, 21]. This finding is in line with published data by Almeida et al. [19]. The intra- and inter-examiner reliability was lower in posterior teeth compared to the results of anterior teeth. This might be explained by the fact, that the restorations in posterior teeth showed more complex clinical situations with a broad variety of deficiencies. With respect to the documented variations between the examiners it must be emphasized that especially researchers need to be theoretically and practically trained in the proper application of the criteria. Future studies which include the updated FDI criteria should integrate a calibration training [5, 6].

This study has some potential strengths and limitations which need to be discussed. One strength worthwhile mentioning is that the selection of images covered a broad spectrum of clinical conditions throughout all domains of the revised criteria set which is difficult to cover in a clinical study set-up. The ten experts and their commitment to improve the criteria is another important feature of this study. The broad experience and expertise of the expert panel was beneficial to the revision of the criteria set. It needs to be noted that the criteria and scoring were constantly improved, so that eventually, mainly outliers of only one score were recorded (Figs. 1 and 2). A weakness, of the study was that the restorations were not evaluated clinically but by means of intraoral photographs. The visual-tactile clinical evaluation of a restoration with a probe and other instruments, e.g. proximal blades and articulation paper, may lead to a more objective scoring. Furthermore, the inspection of the restored tooth from different angles and perspectives enhances the clinical evaluation which is not possible when intraoral photographs were the only evaluation tool [19]. The latter aspect is especially relevant for those criteria which are not scorable on intraoral images, e.g. “occlusion and wear,” “proximal contact point,” and “postoperative hypersensitivity/pulp status.” Consequently, these criteria were not included in the reliability study. Also, the evaluation of aesthetic properties on photographs might be different compared to the clinical evaluation and may influence the assessment of surface lustre, surface texture, and colour [18, 19, 22]. Nevertheless, intraoral photographs seem to be useful tool for the evaluation of dental restorations [18, 22, 23].

Another weakness is the low sample size of 49 photographs and the focus on tooth-coloured restorations only. The inclusion of more images and restoration materials would have increased the validity of the study but would also have involved more work for the experts as well as extended evaluation sessions. Therefore, it was decided to limit the sample size but increase the number of more difficult cases to represent a broad spectrum of restoration deficiencies. In this context, it has to be pointed out that a rigorous testing would include two examinations per each evaluation round to better determine the intra- and inter-examiner reliability. The requirement of a second examination was not met due to the time resources of the experts. Furthermore, it has to be mentioned that there was an unbalanced distribution of restoration deficiencies across the selected clinical cases which resulted in a higher number of sufficient scores in a few categories. This may have influenced the Kappa values which justified the inclusion of the percental agreement, modified Bland/Altman plots, and the binominal logistic regression model using backward elimination. The consistent and complete reporting of these statistical data should be assessed as valuable and may improve the comparability between previous and future studies.

## Conclusions

The overall reliability of the revised FDI criteria set for the evaluation of direct and indirect dental restorations was steadily increased up to the final version. However, significant differences were documented for some of the examiners, categories, and tooth type. Training and calibration are required to ensure reliable application of the evaluation criteria.

## Supplementary Information

Below is the link to the electronic supplementary material.

Supplementary file1 (PDF 2659 KB)
