# Supplementary material for: Evaluation of direct restorations using the revised FDI criteria: results from a reliability study
Source: Clin Oral Investig. 2022 Nov 18;27(4):1519–28. doi: 10.1007/s00784-022-04771-9 (PMC10102028; doi:10.1007/s00784-022-04771-9)
Supplement: Supplementary file 1 — Supplementary file1 (PDF 2659 KB) [file 784_2022_4771_MOESM1_ESM.pdf]

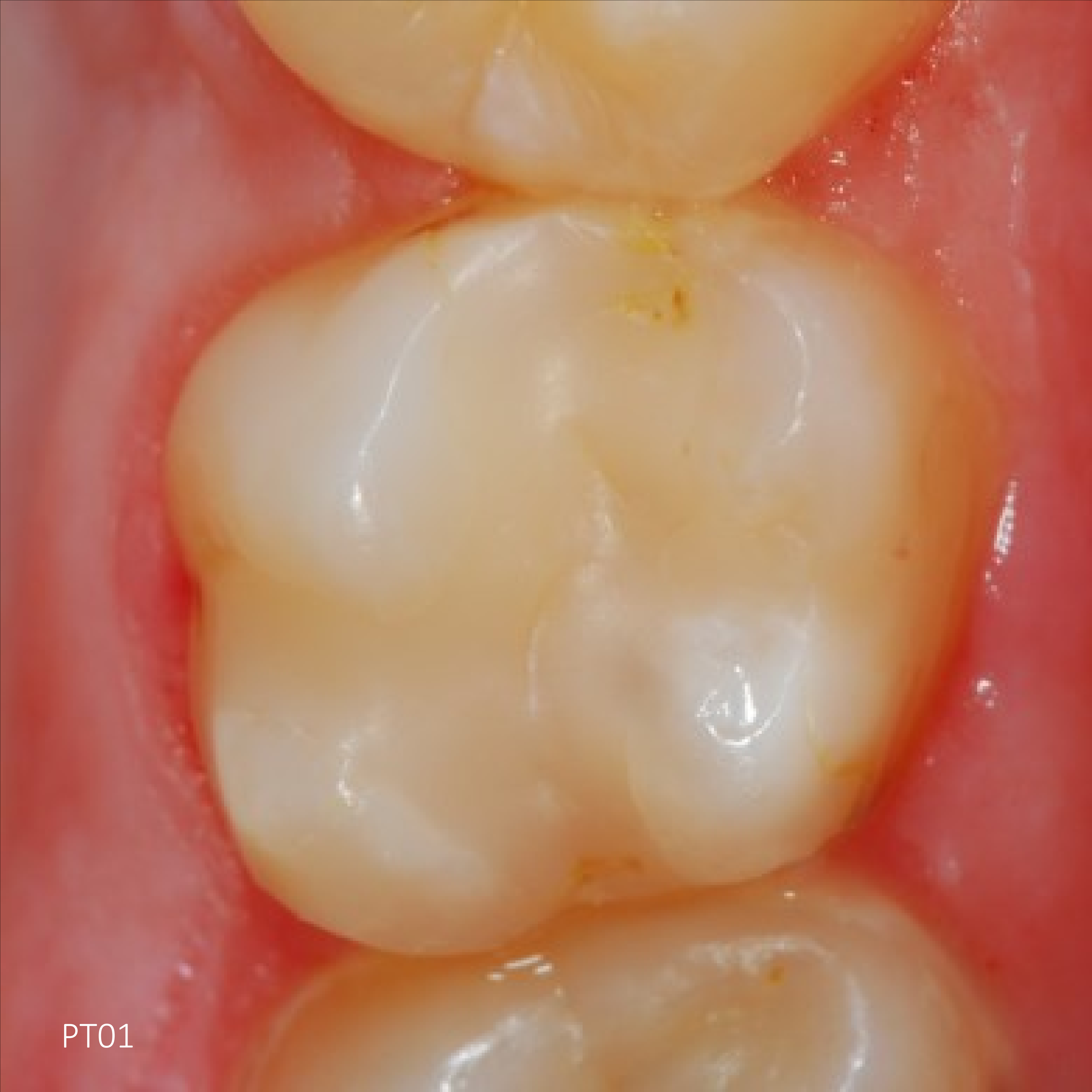
PT01

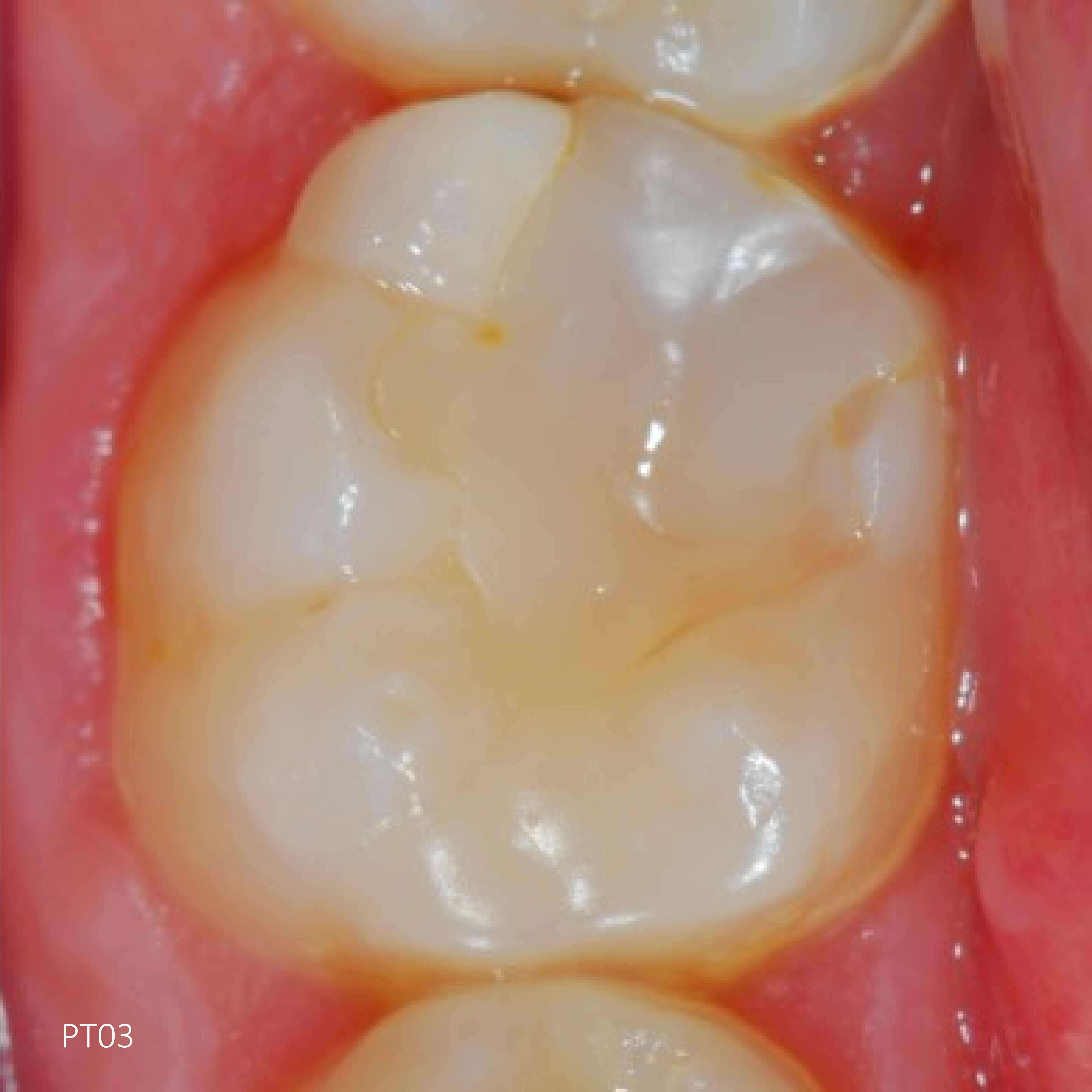
PT03

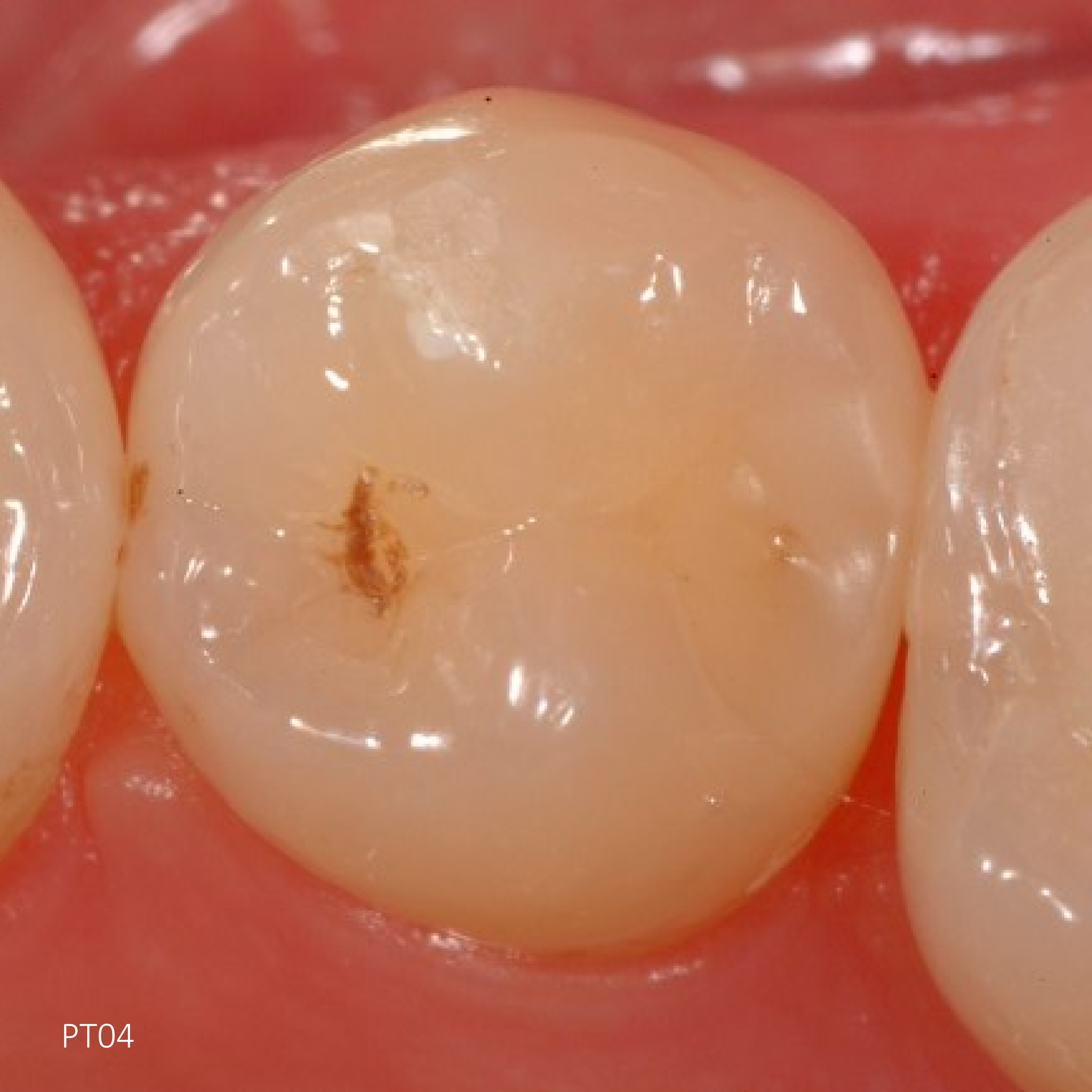
PT04

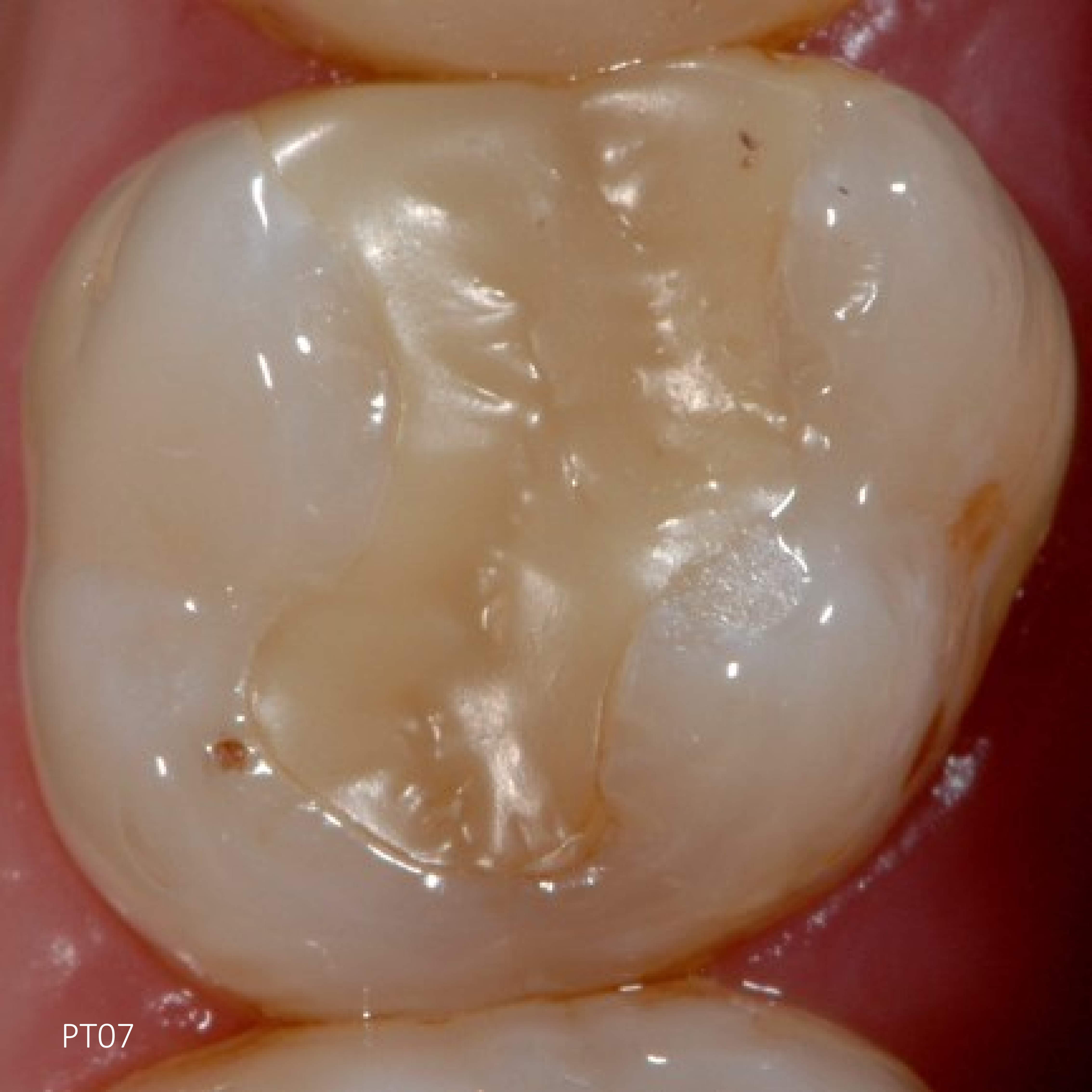
PT07

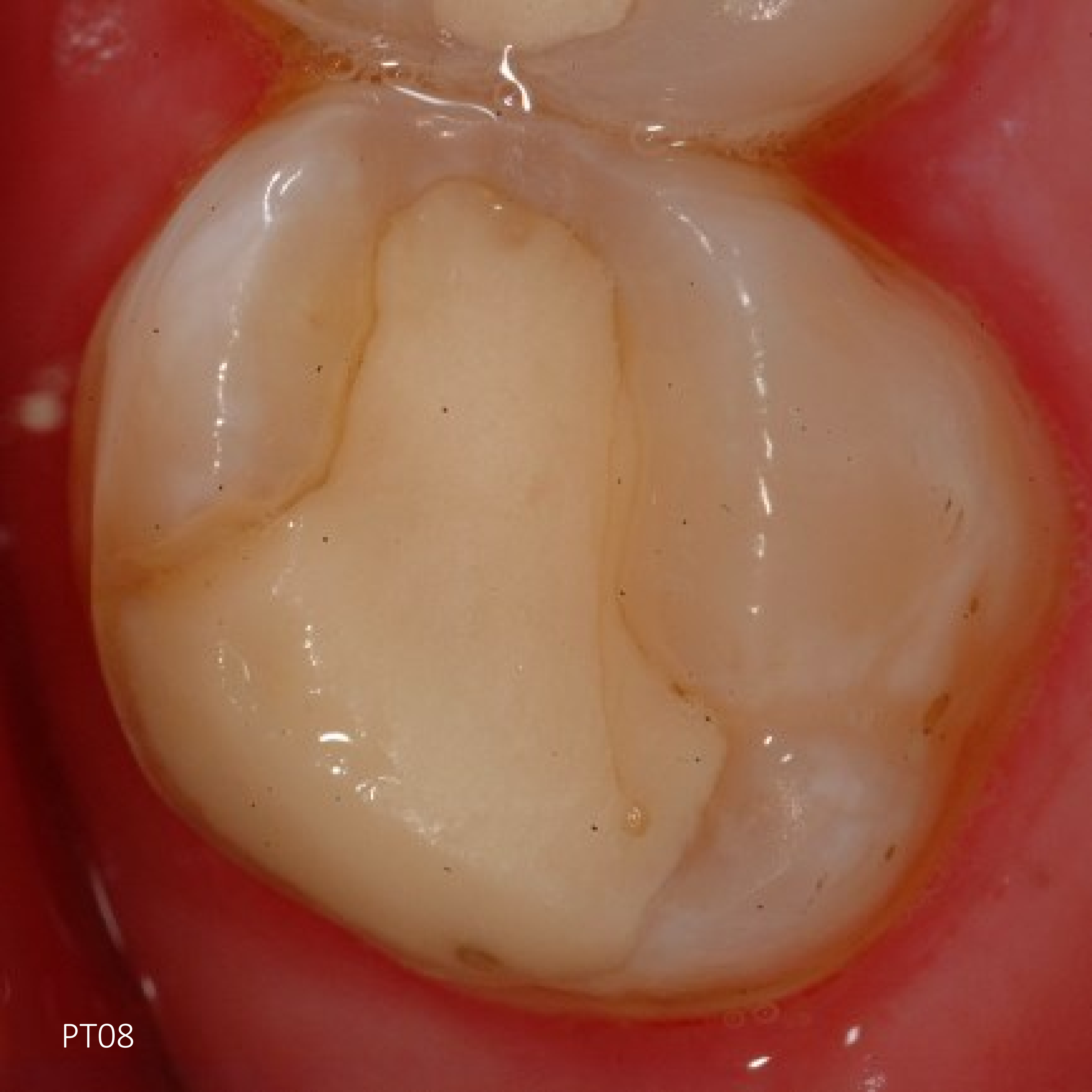
PT08

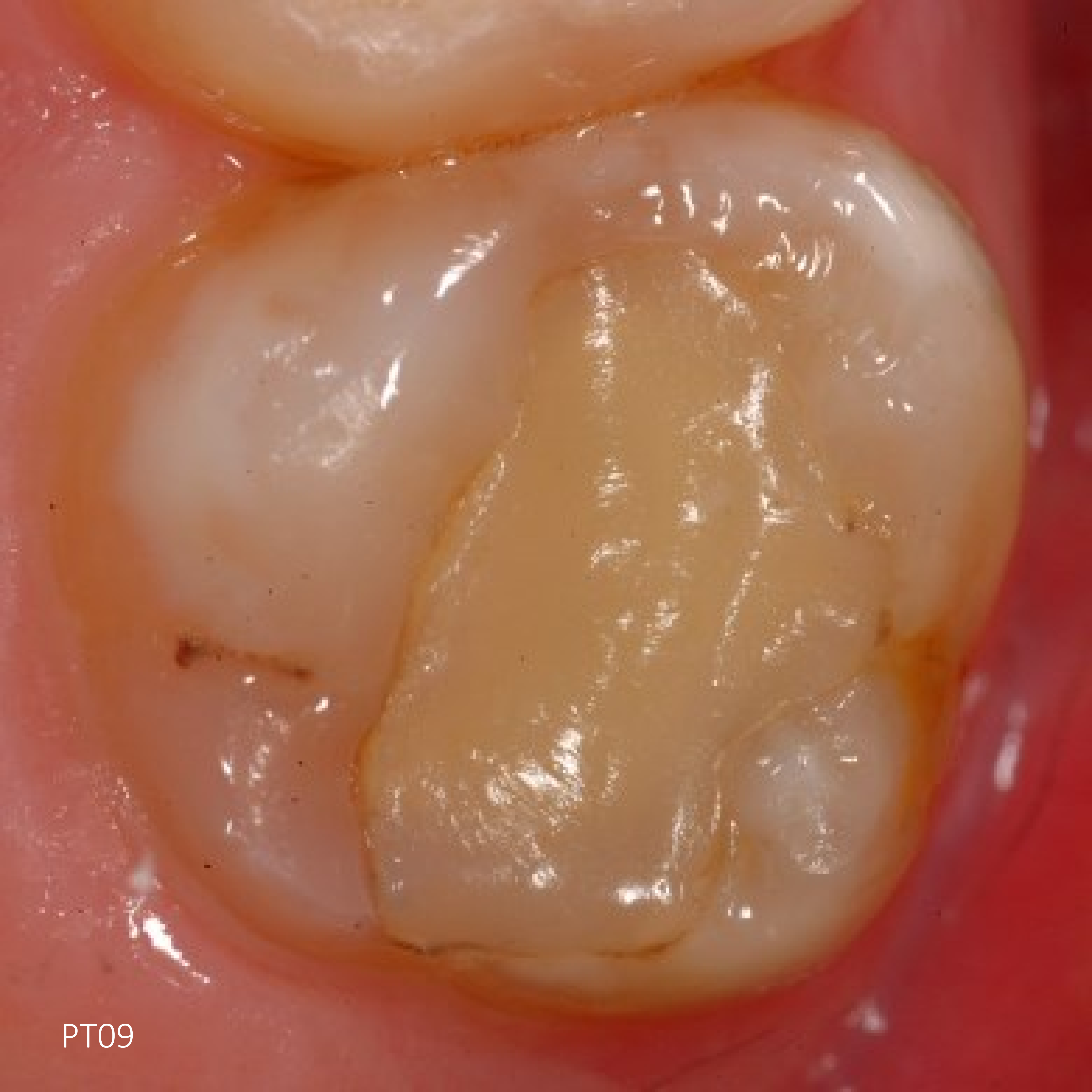
PT09

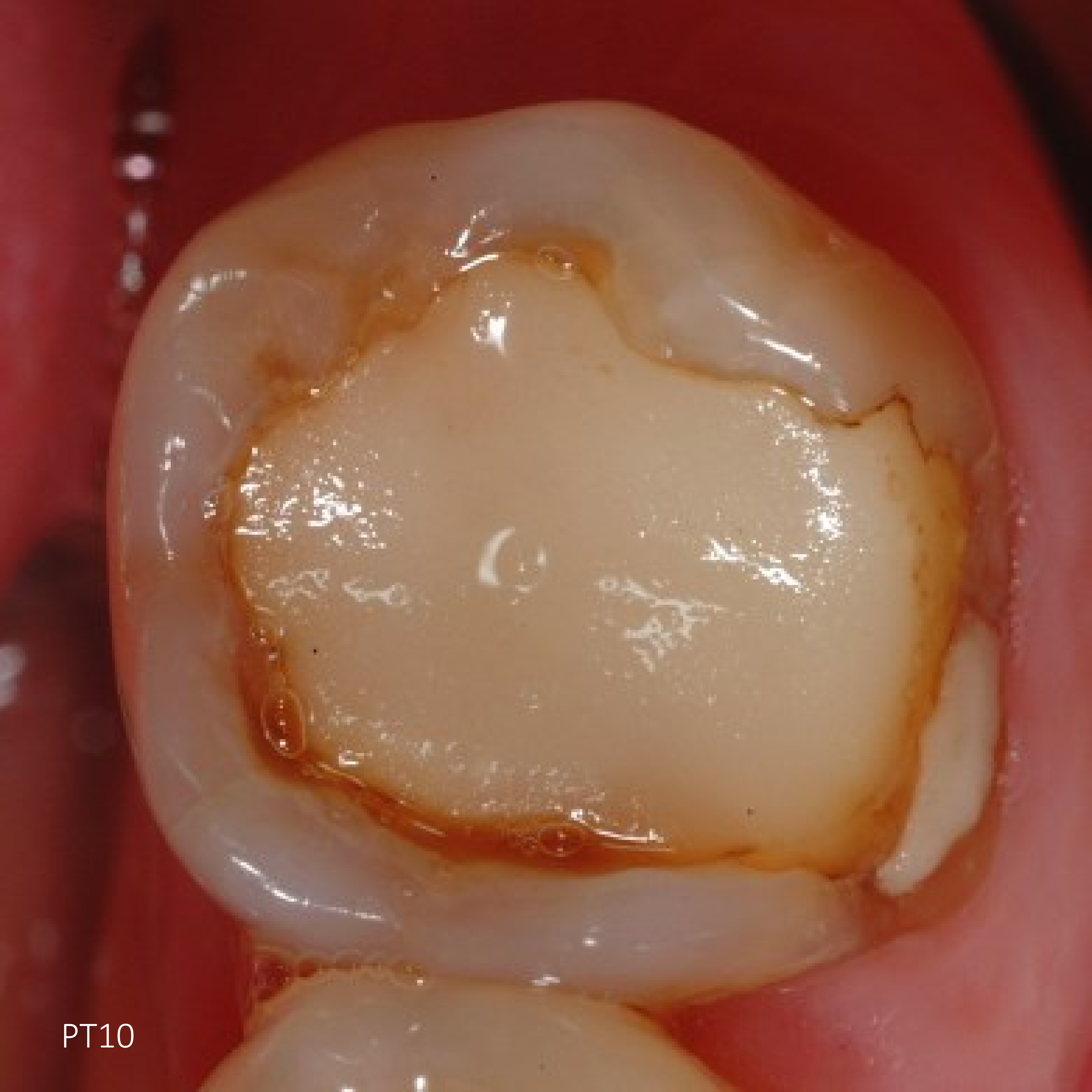
PT10

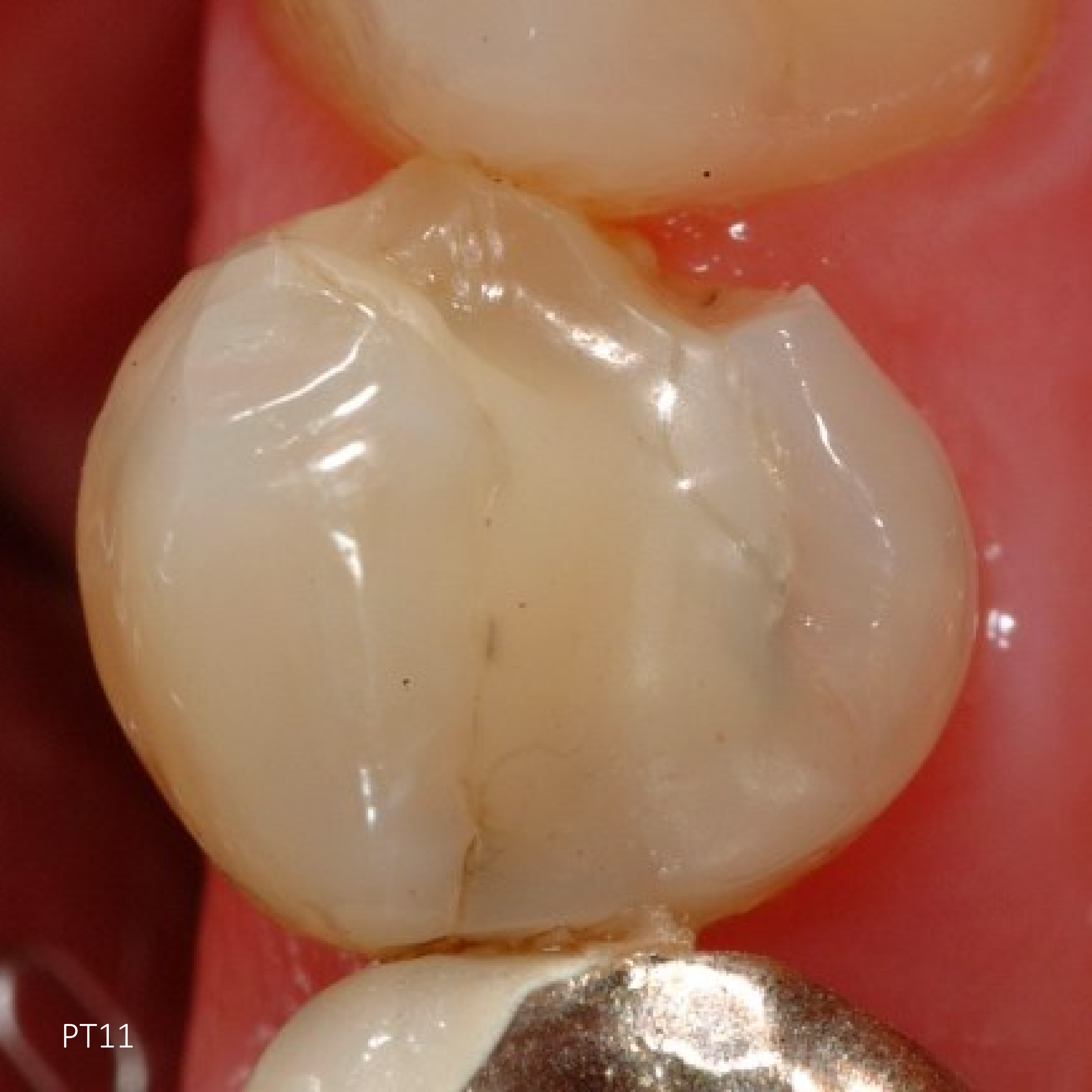
PT11

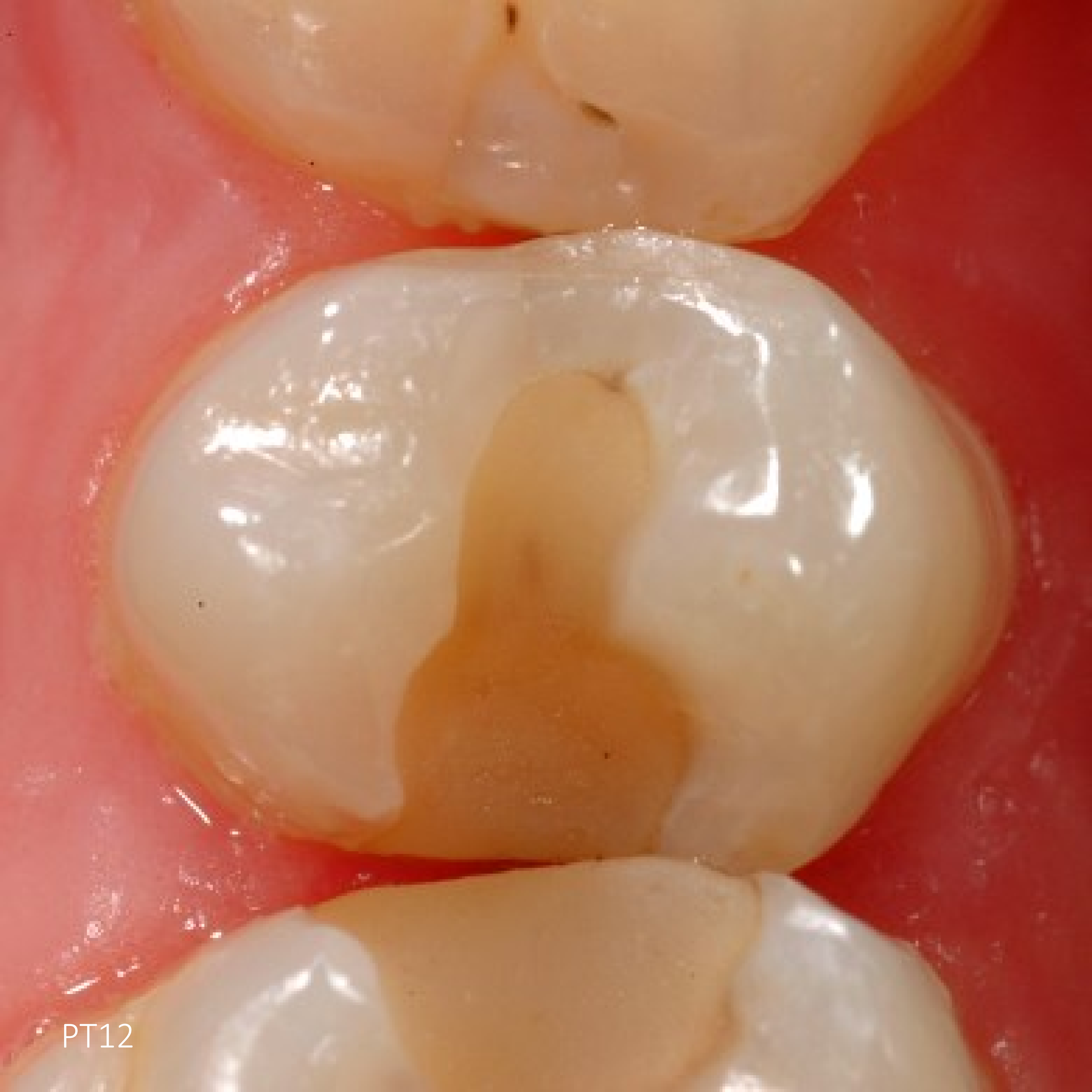
PT12

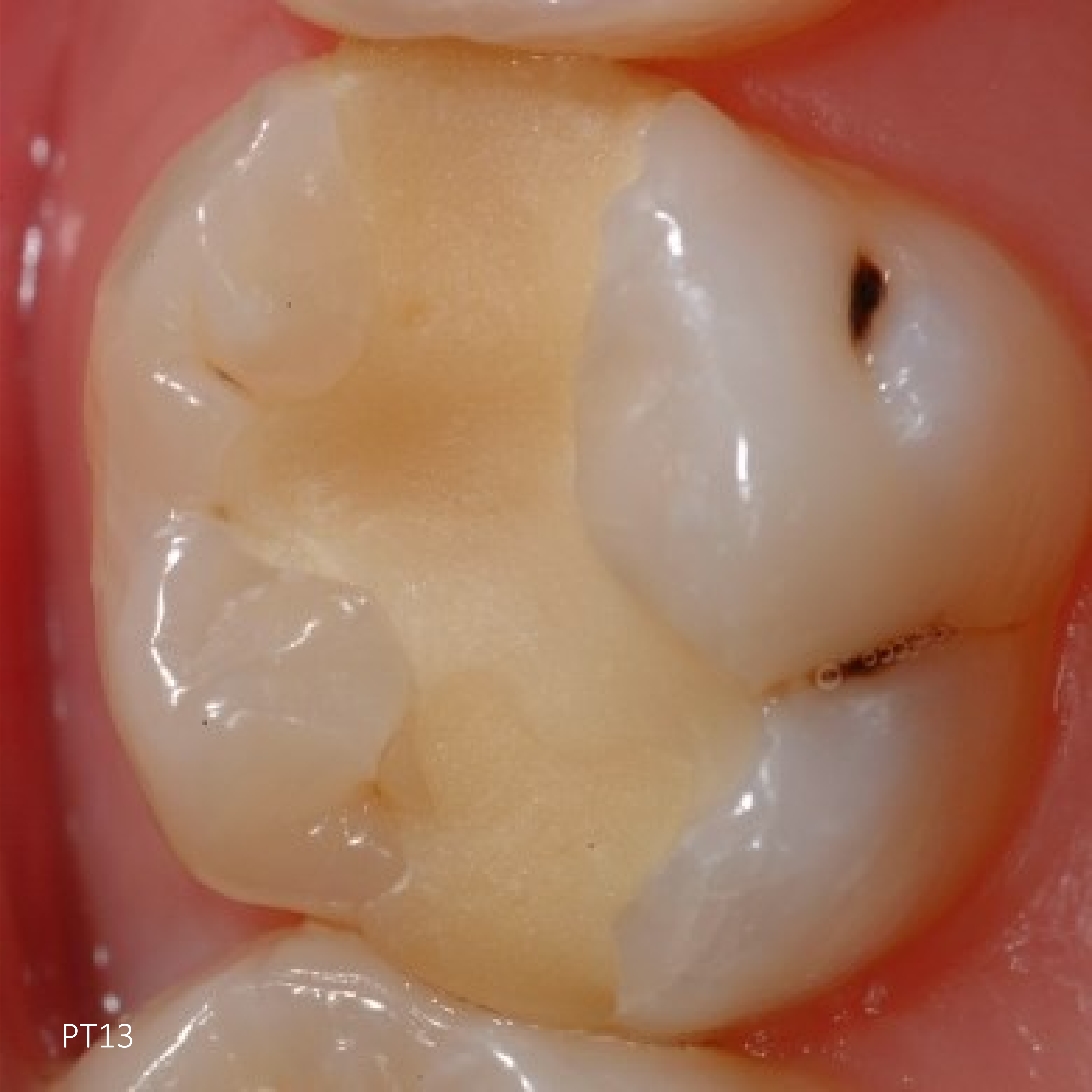
PT13

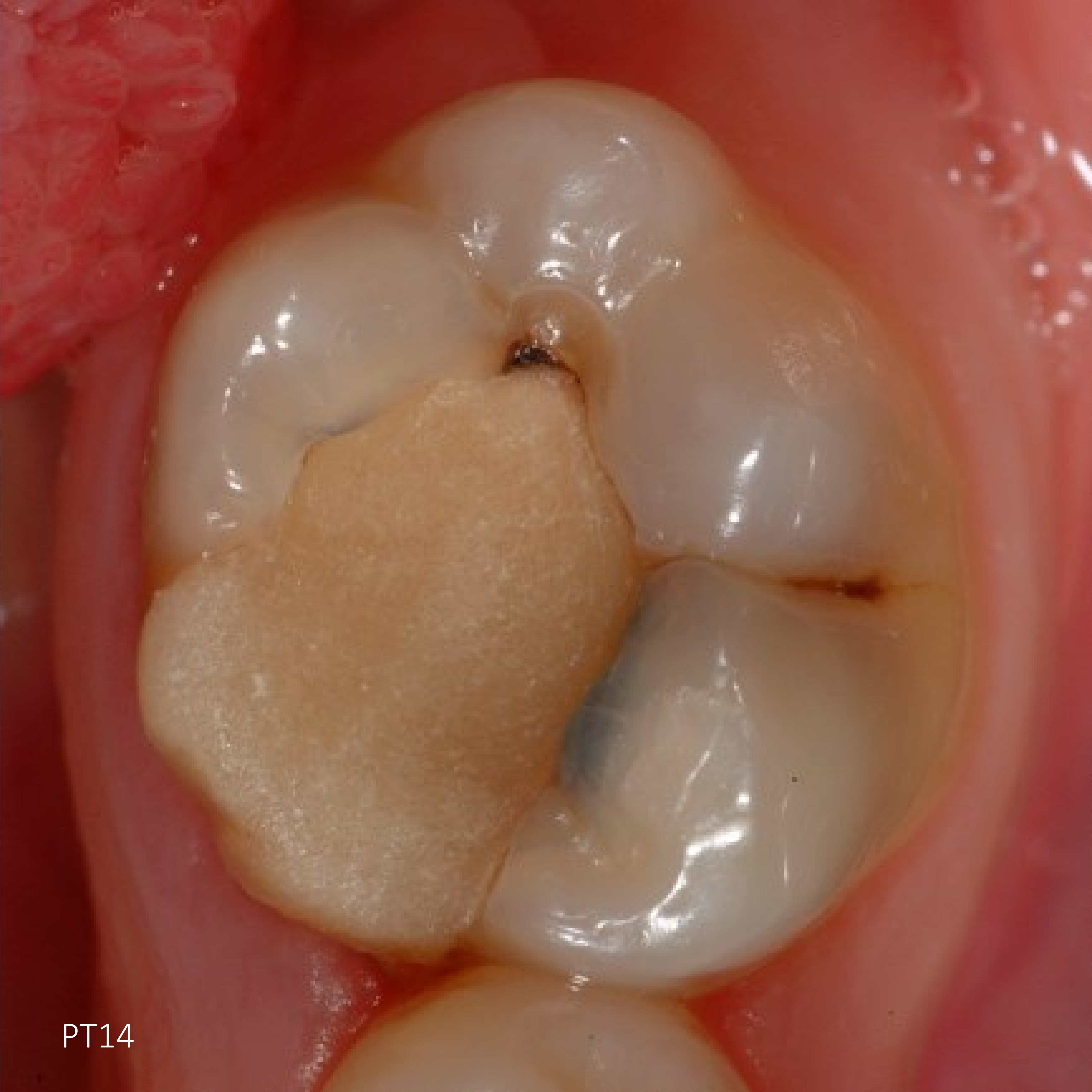
PT14

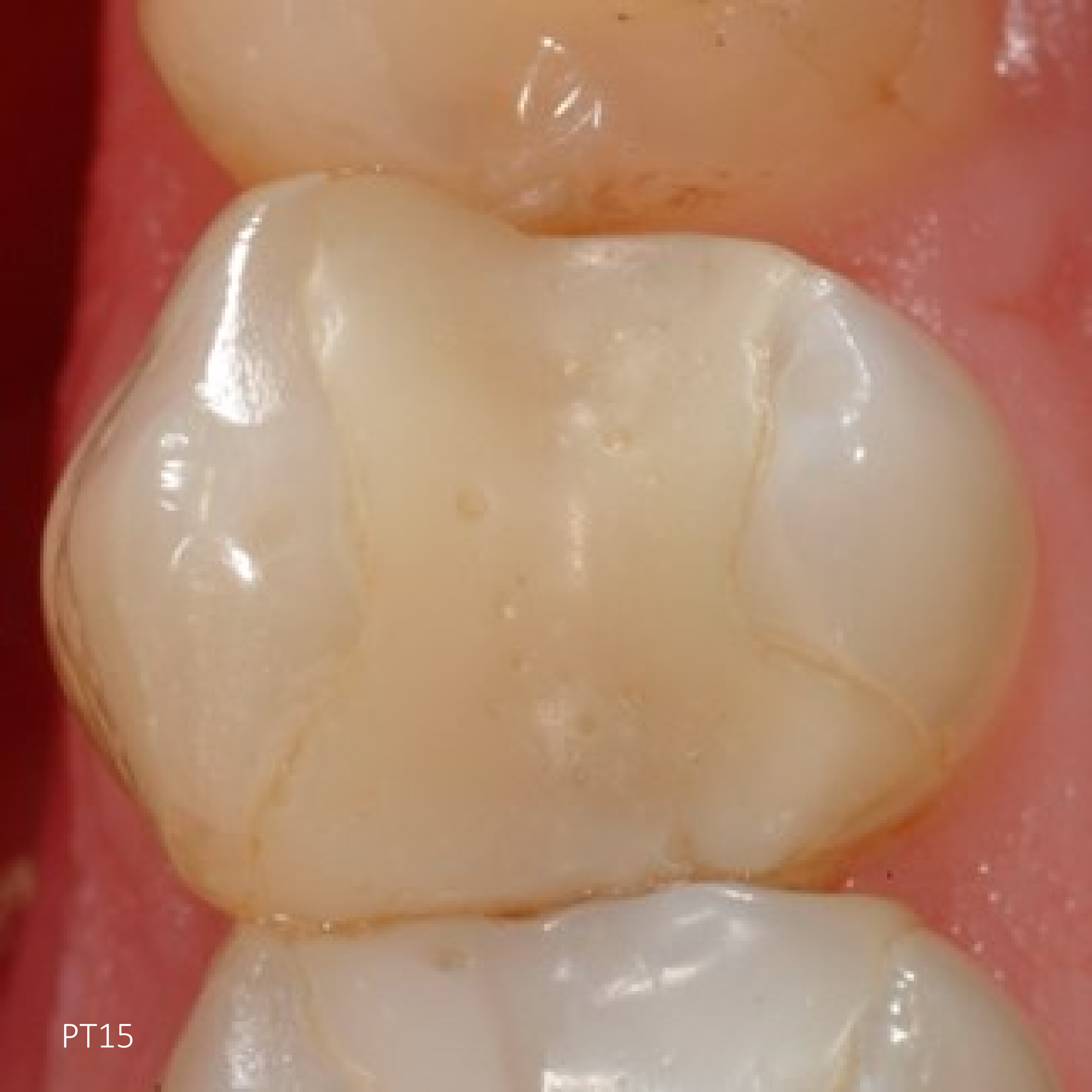

PT15

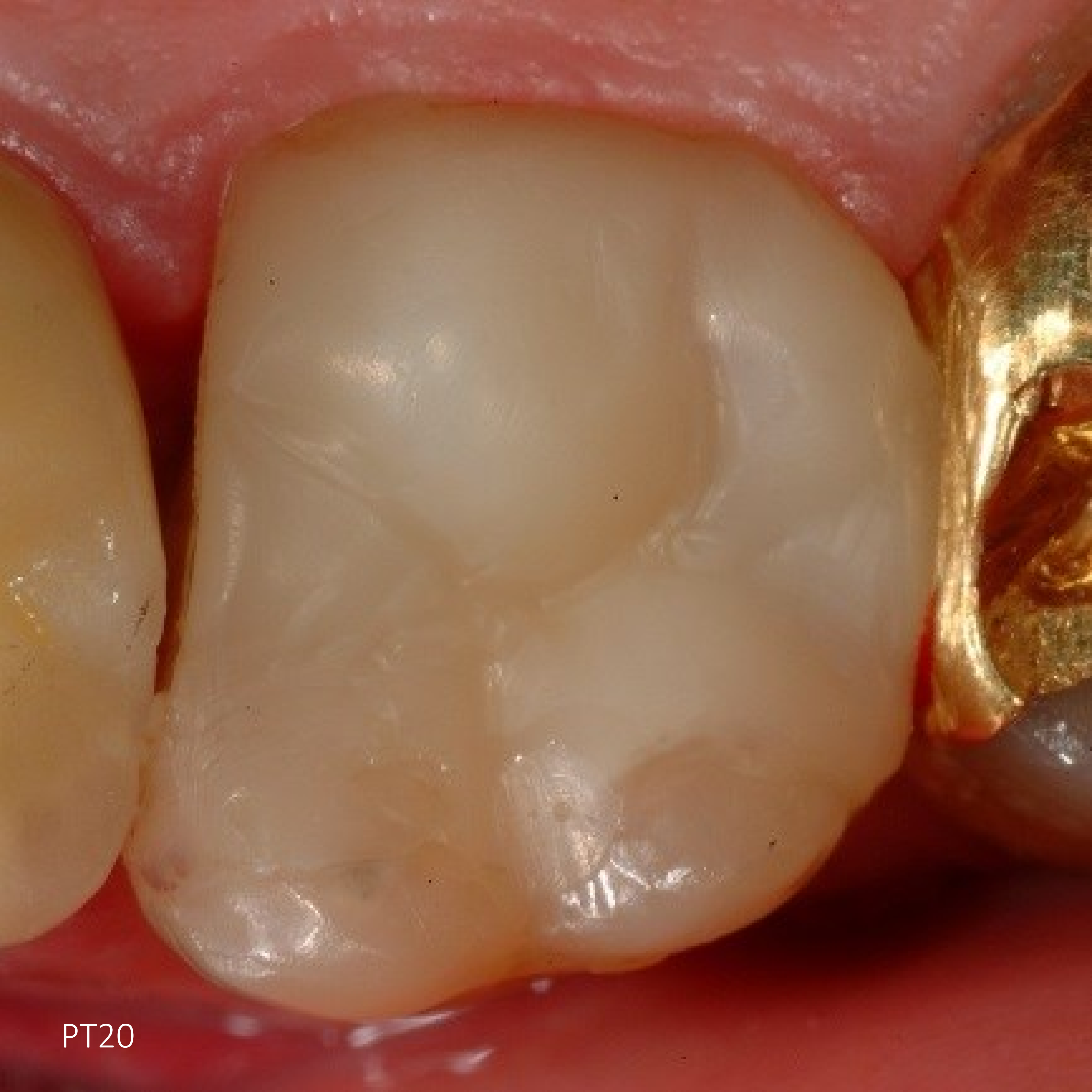
PT20

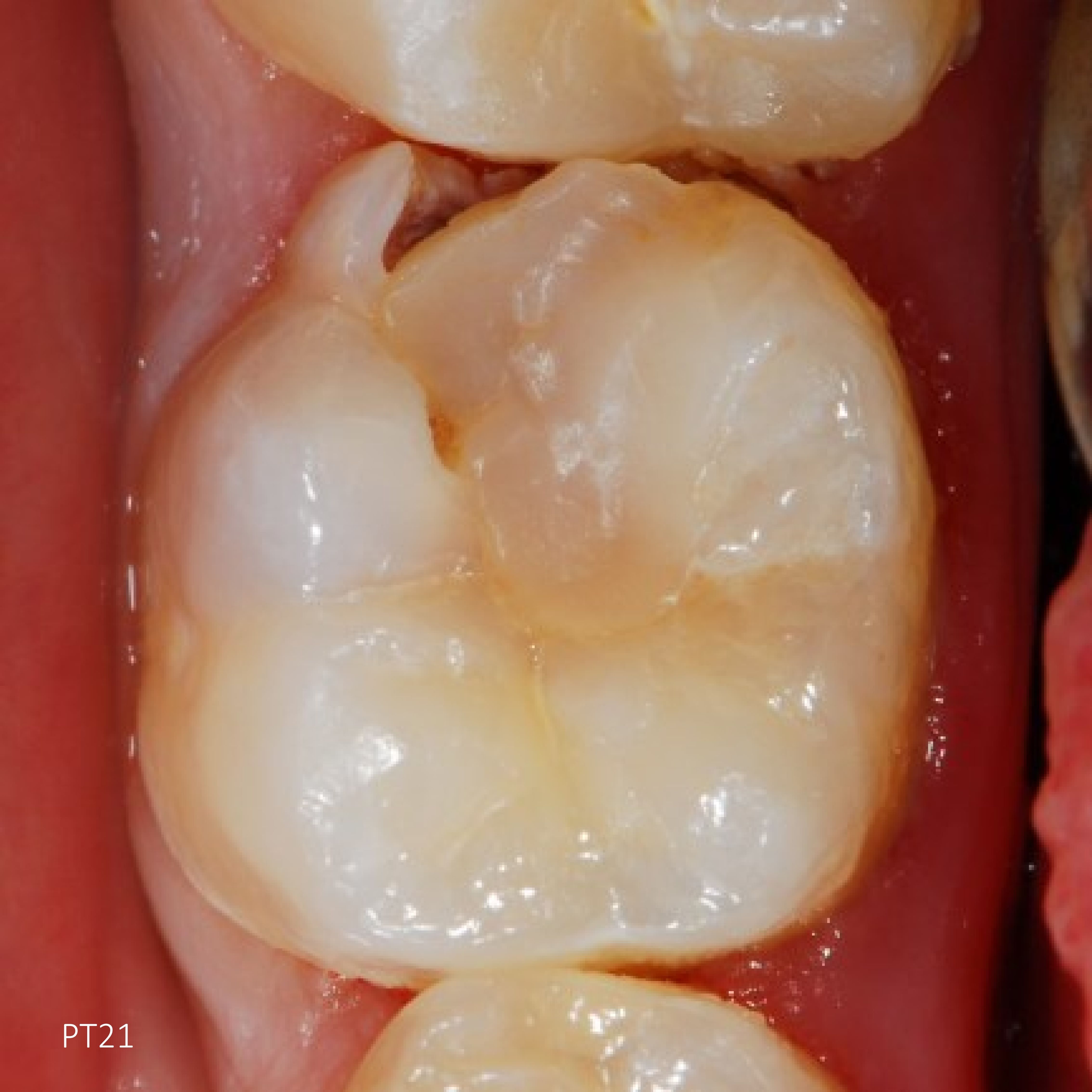
PT21

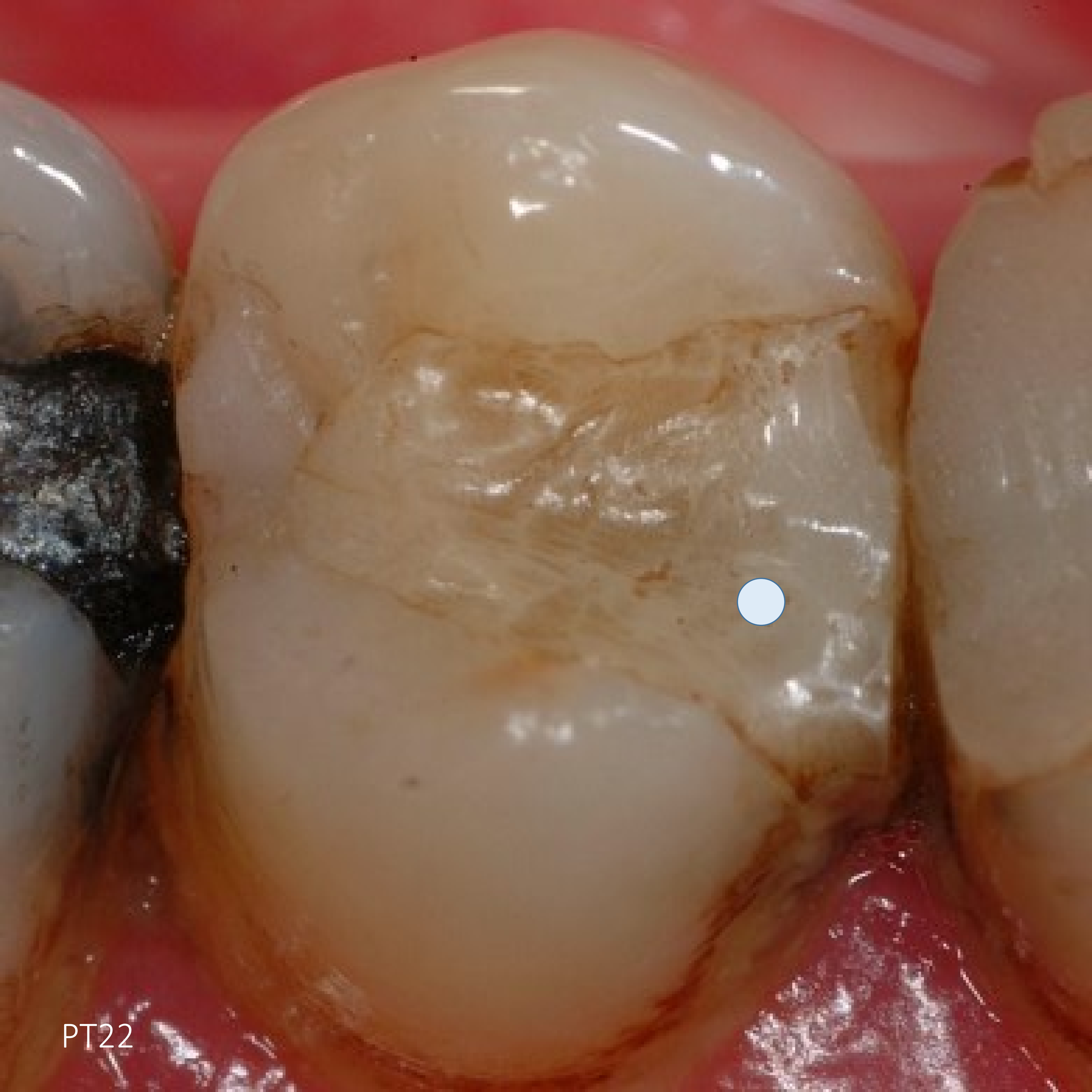
PT22

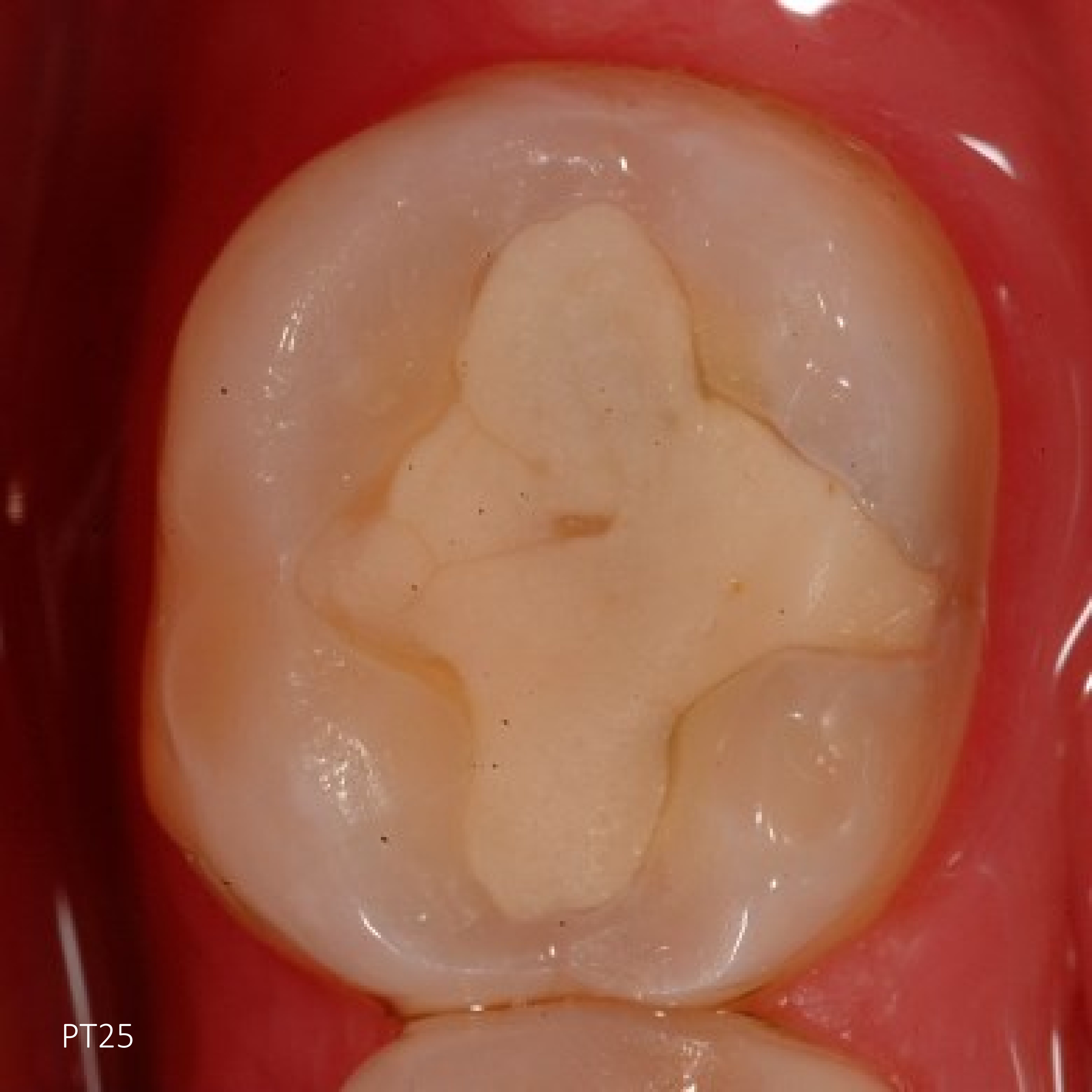
PT25

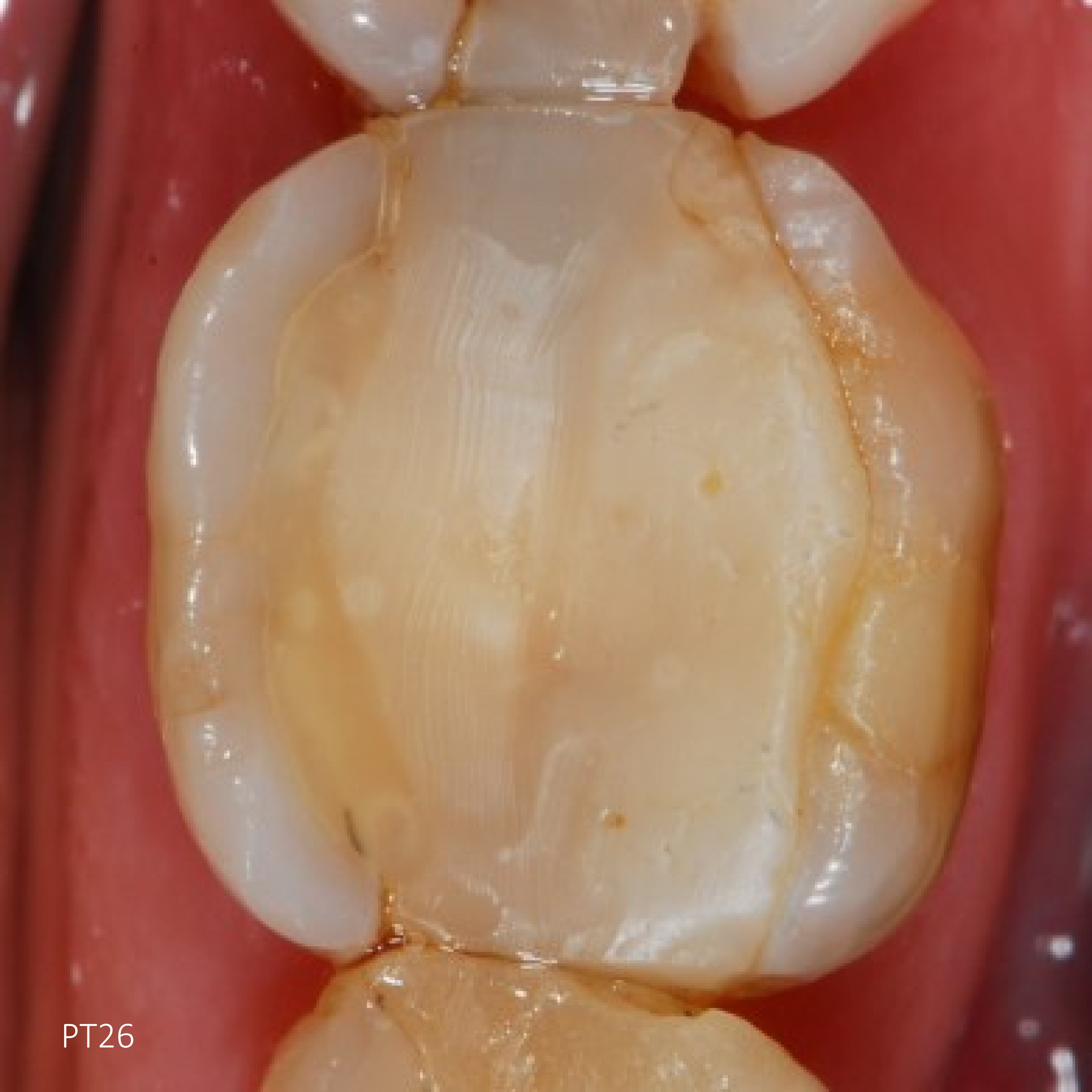
PT26

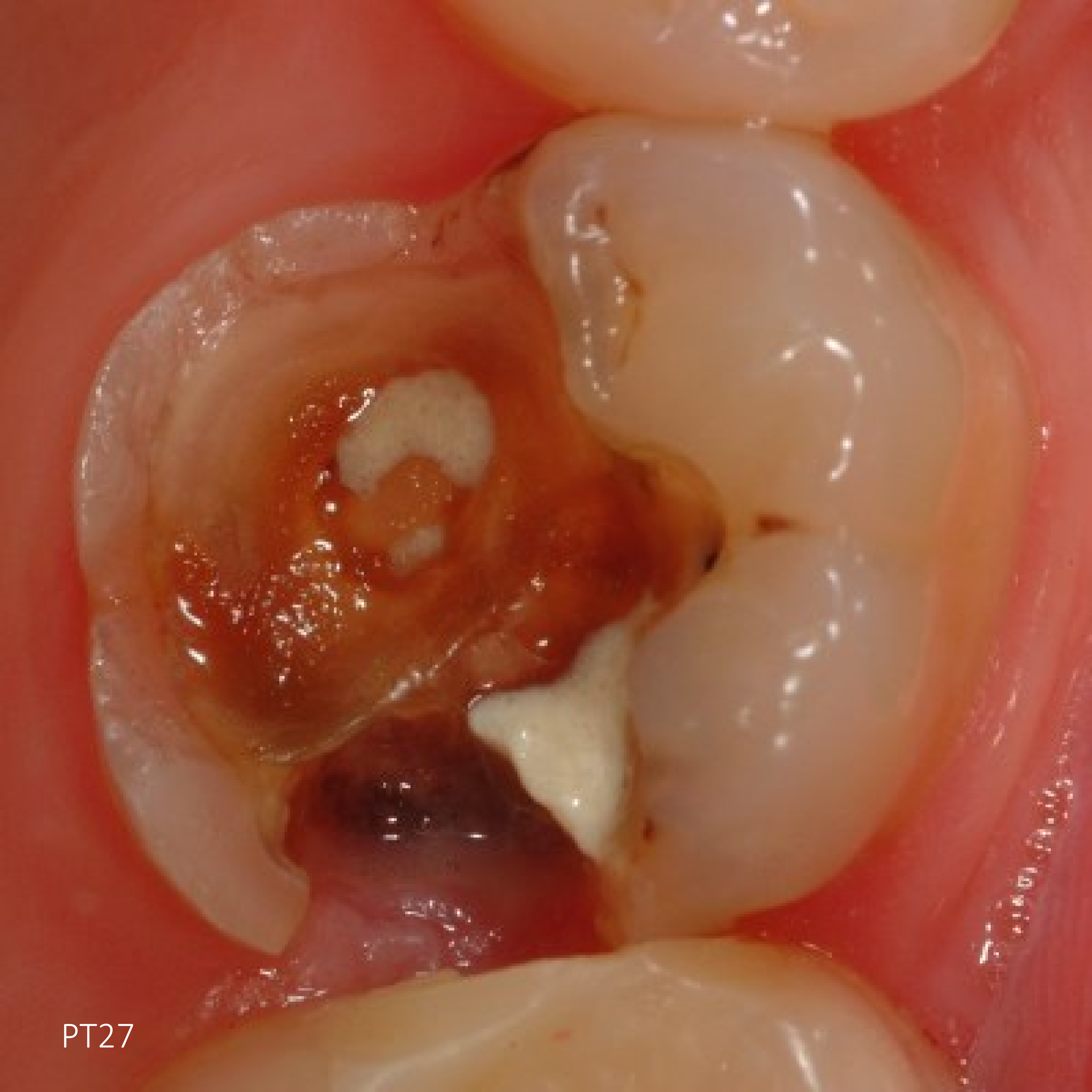
PT27

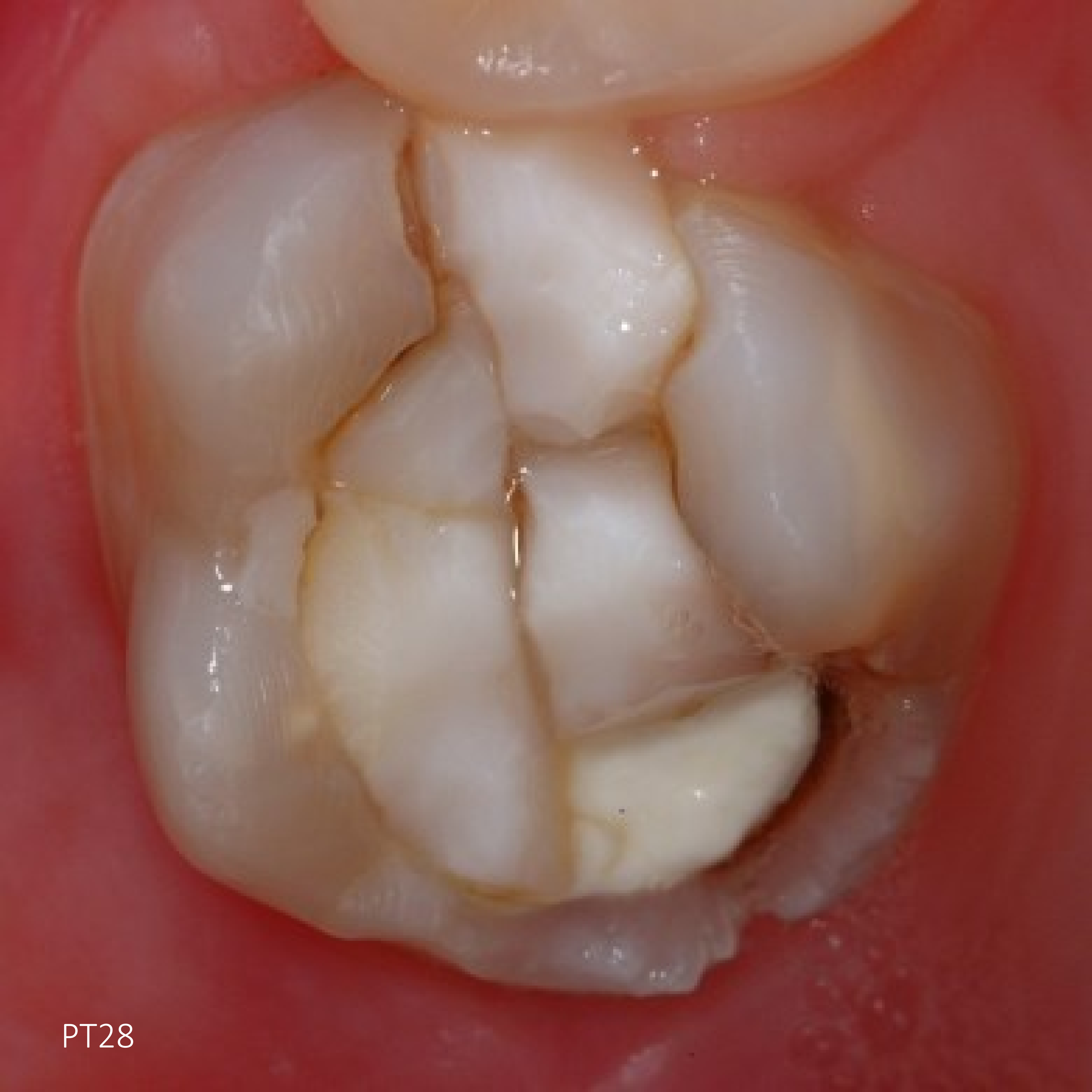
PT28

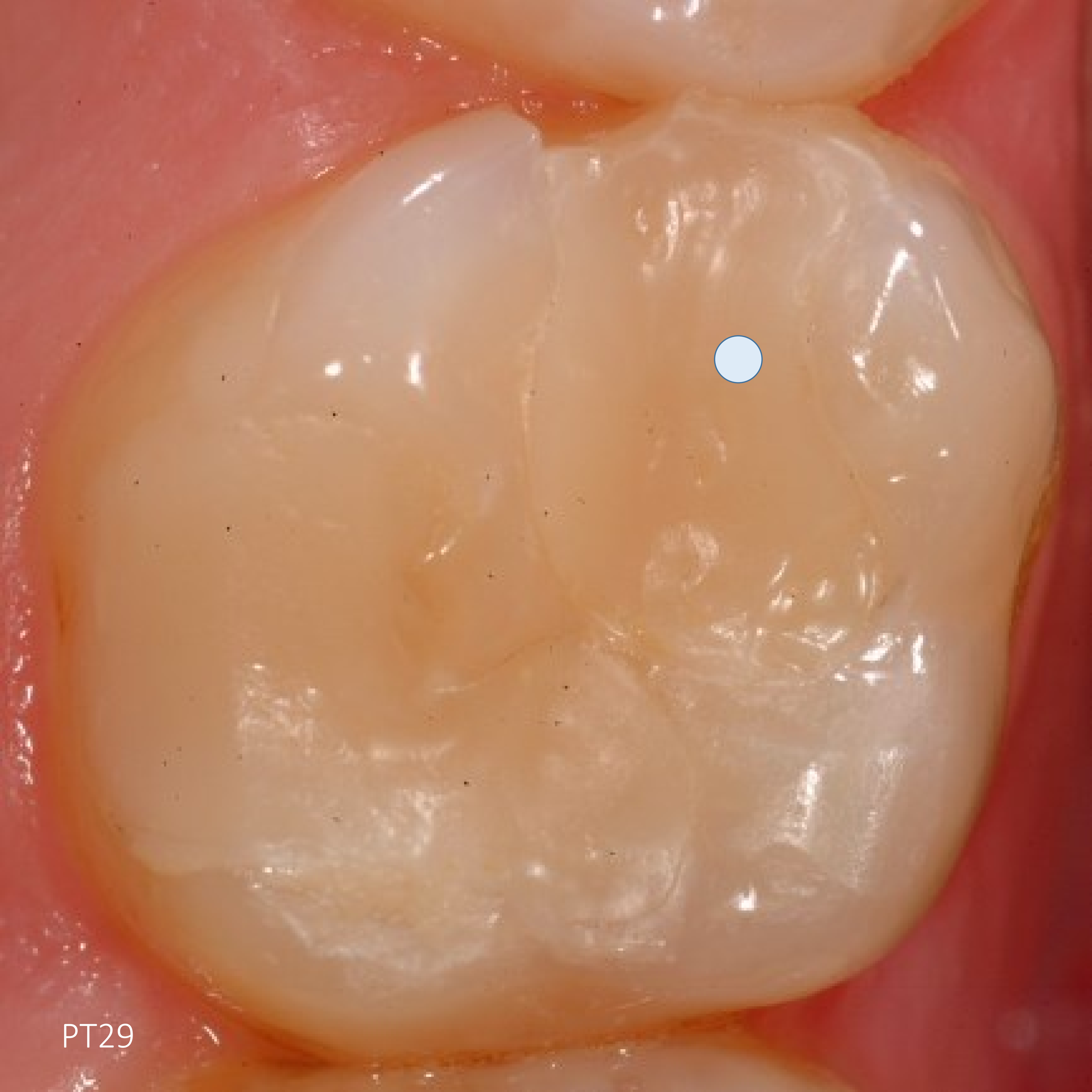
PT29

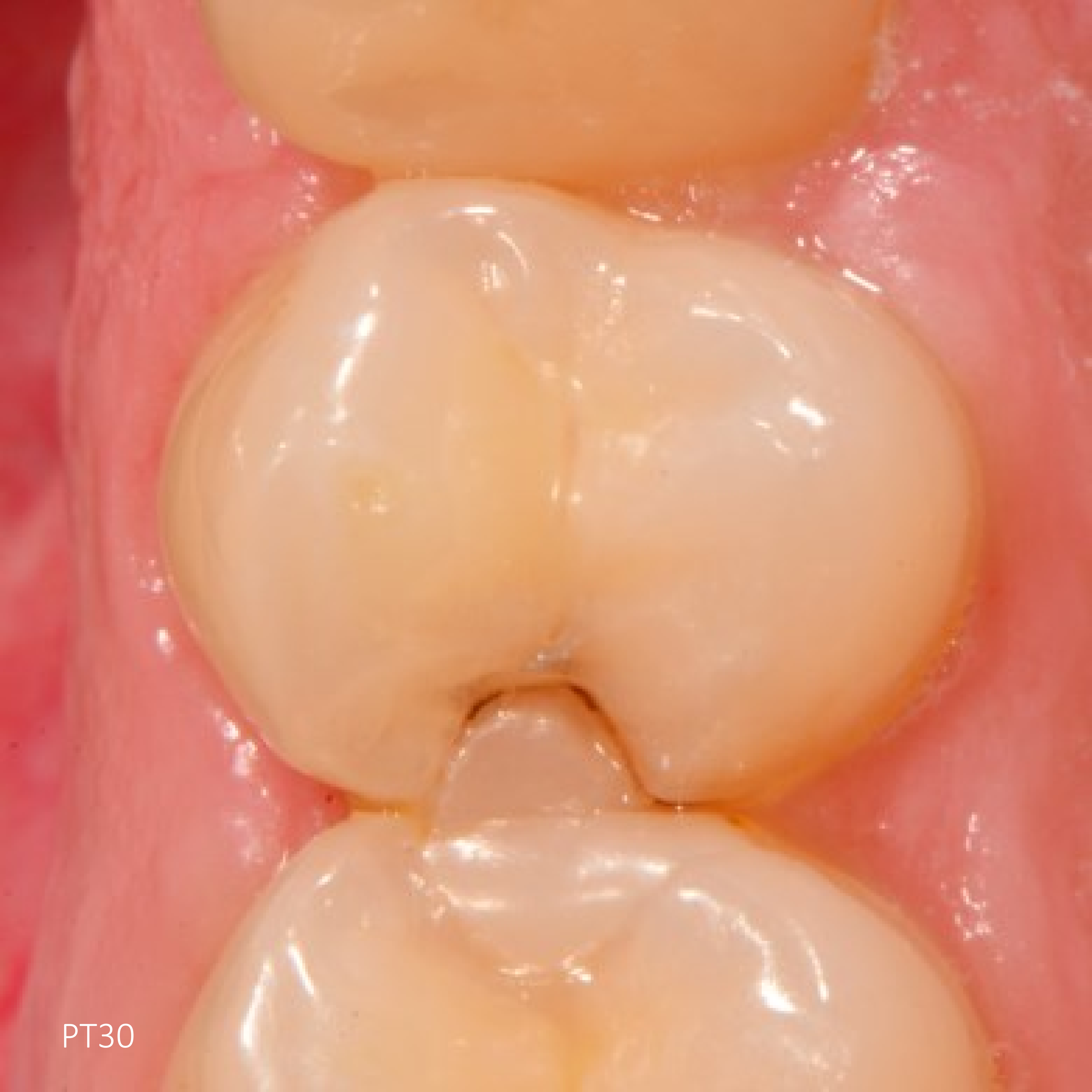
PT30

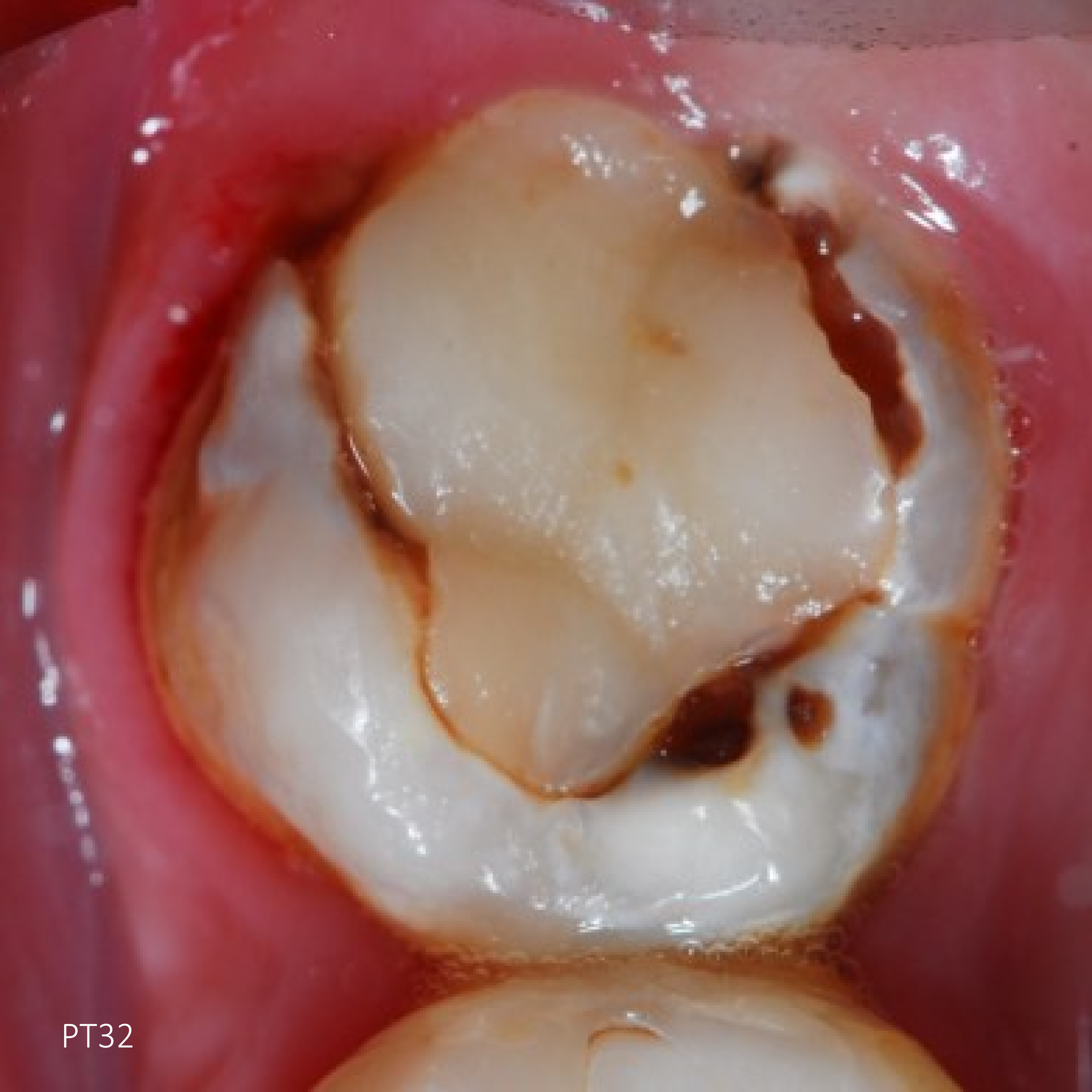
PT32

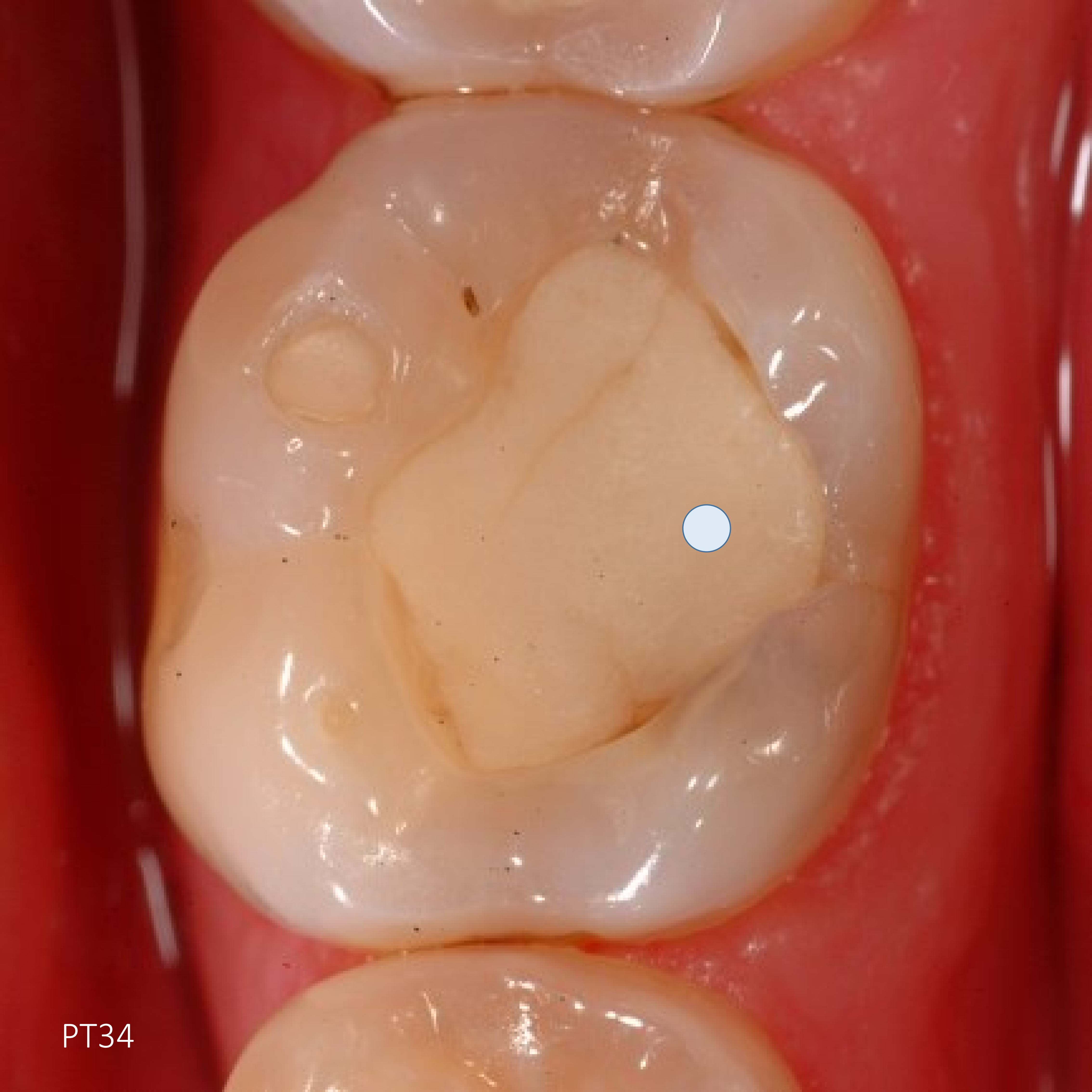

PT34

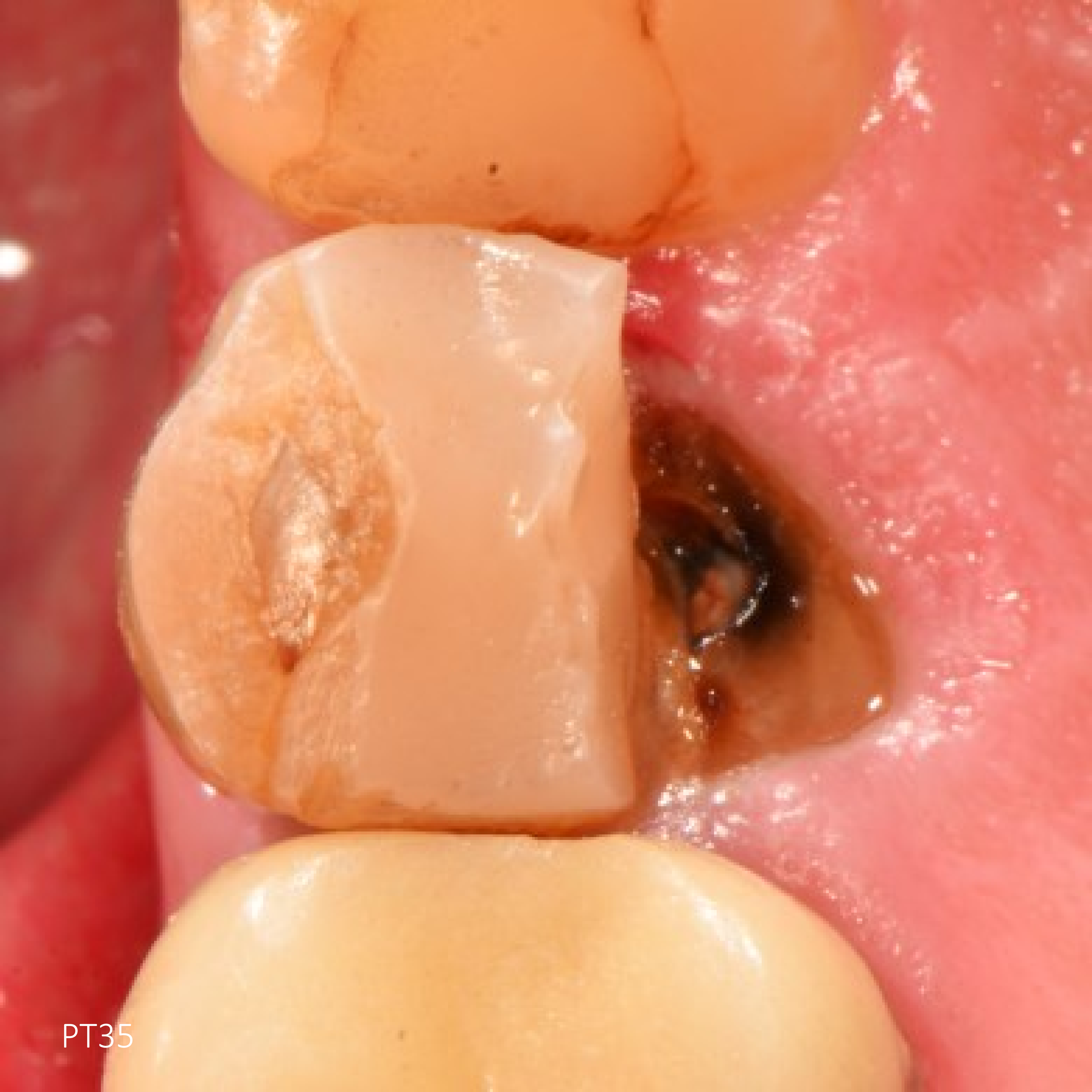
PT35

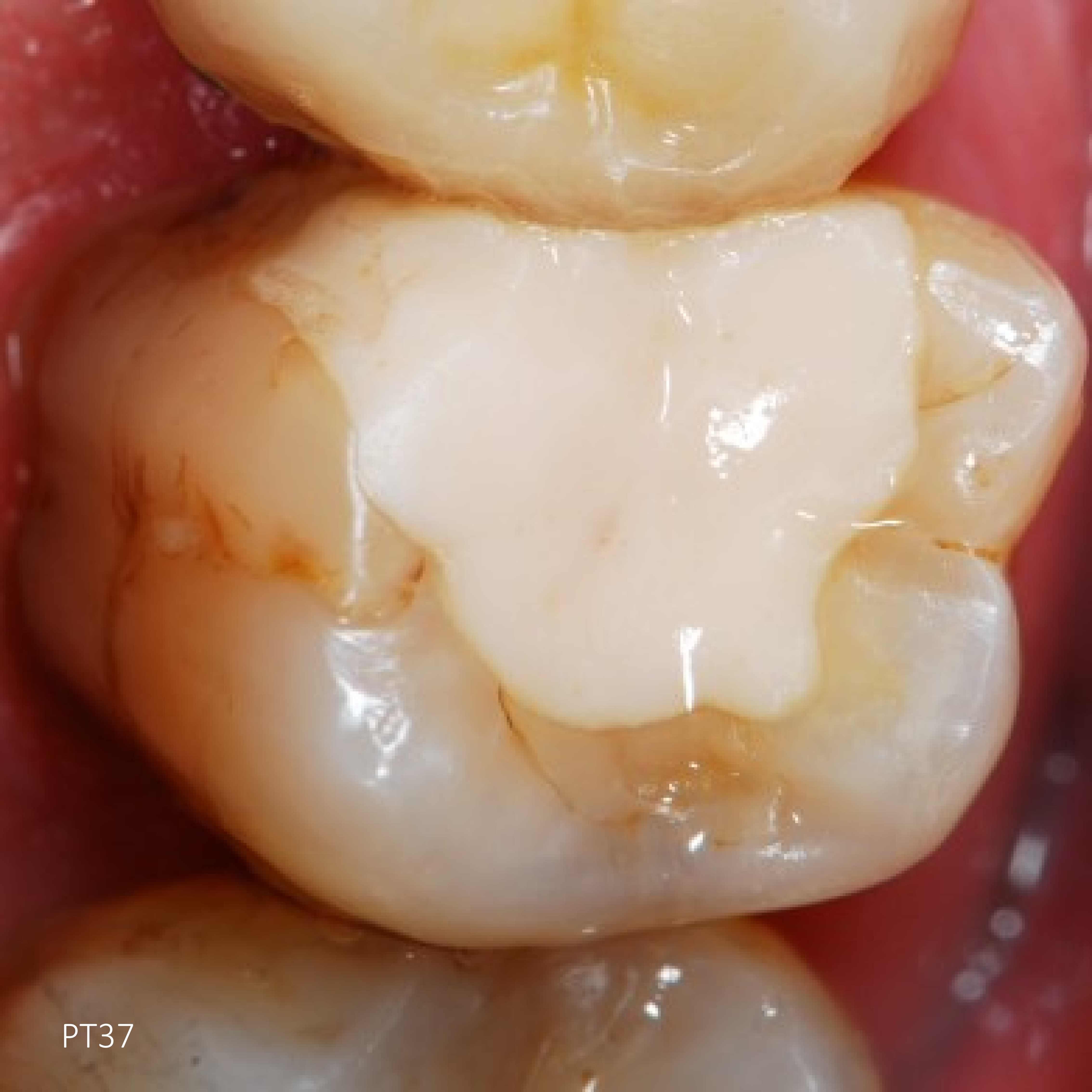
PT37

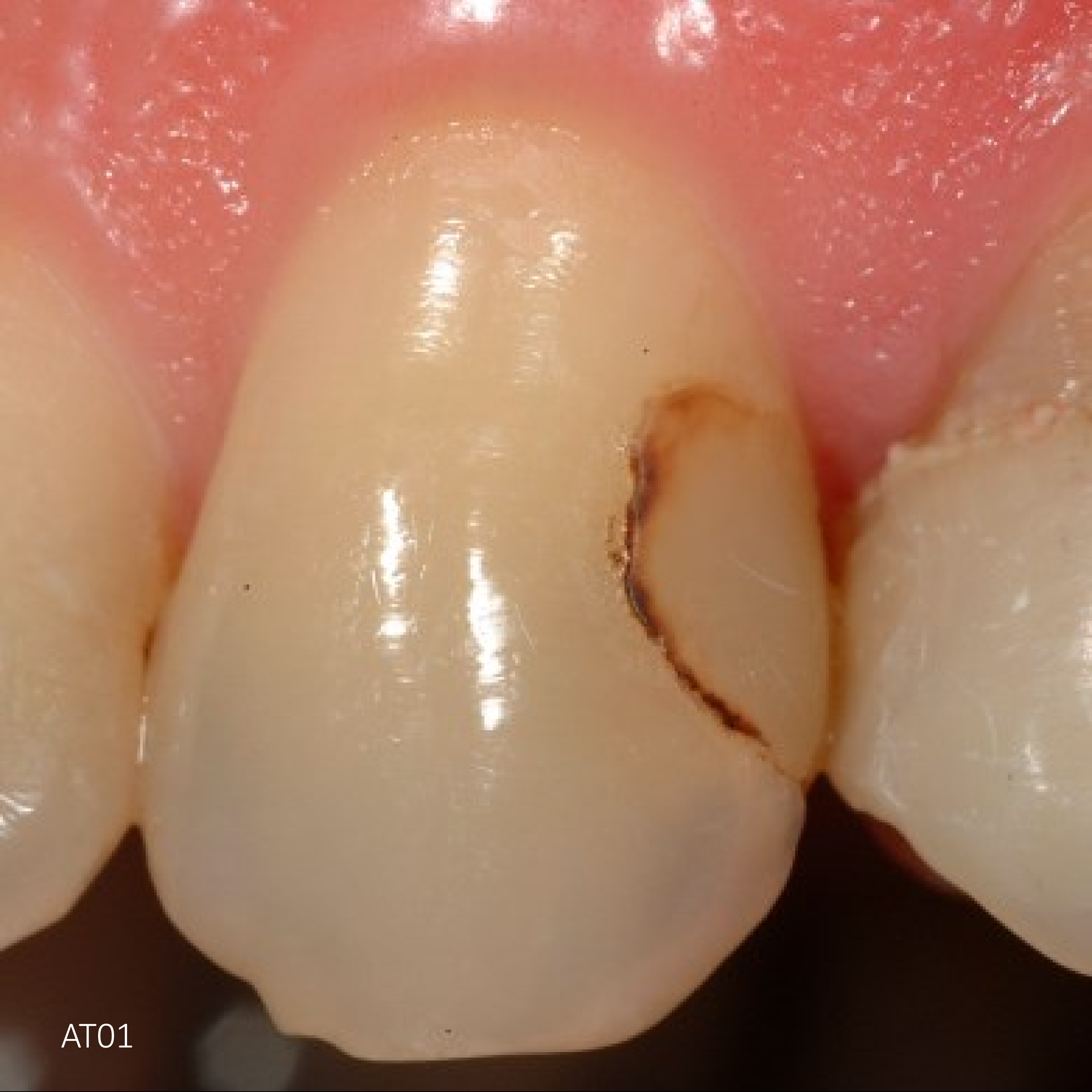
AT01

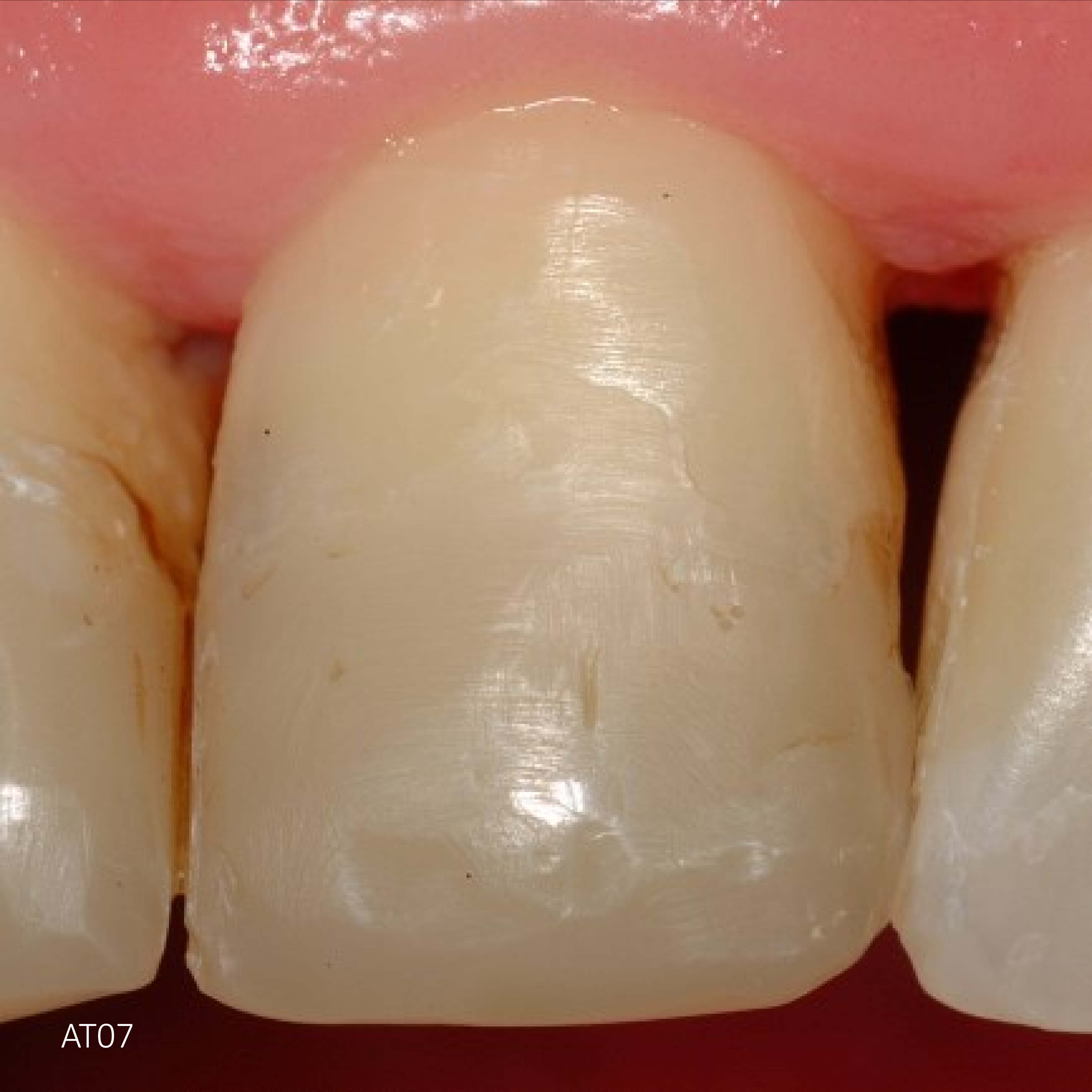
AT07

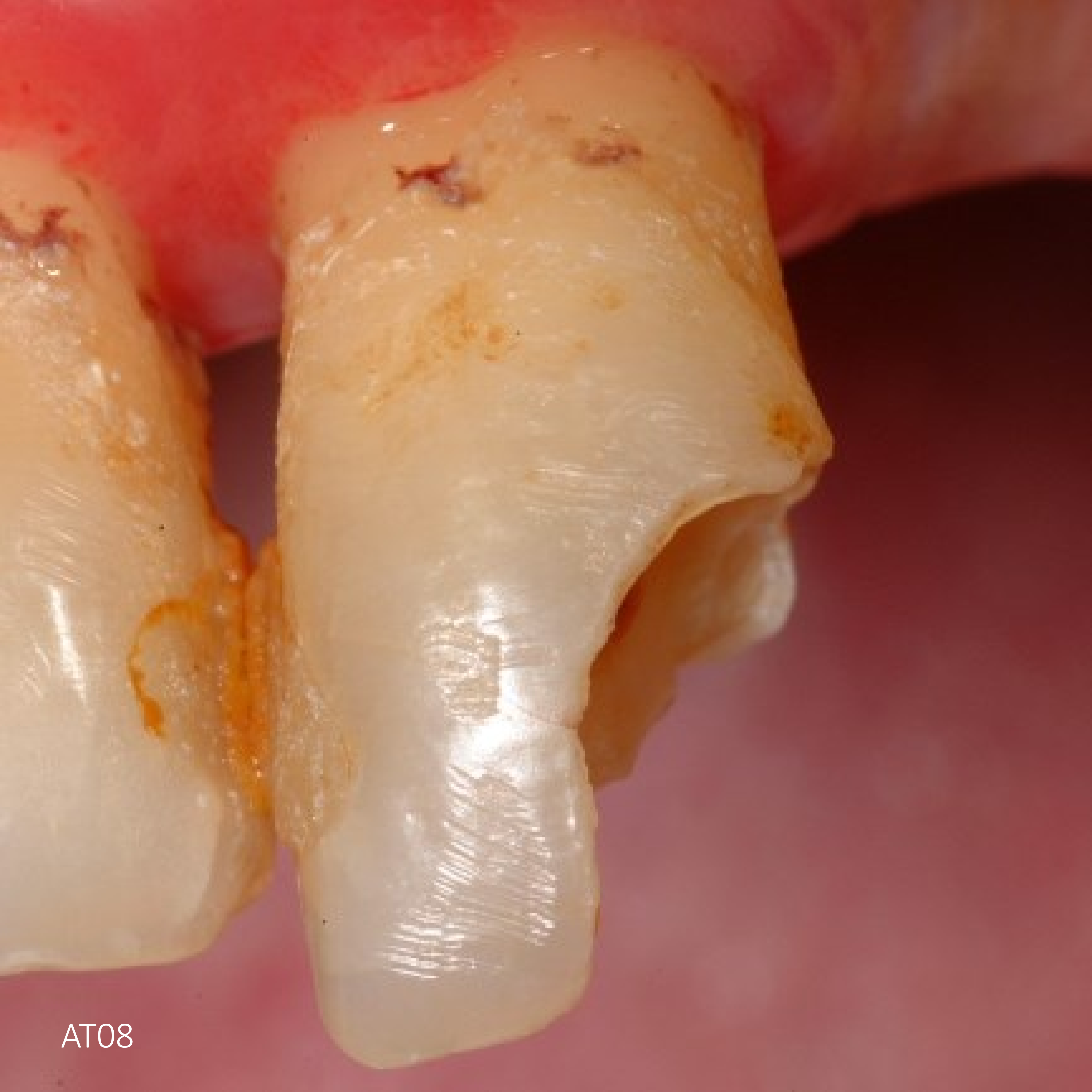

AT08

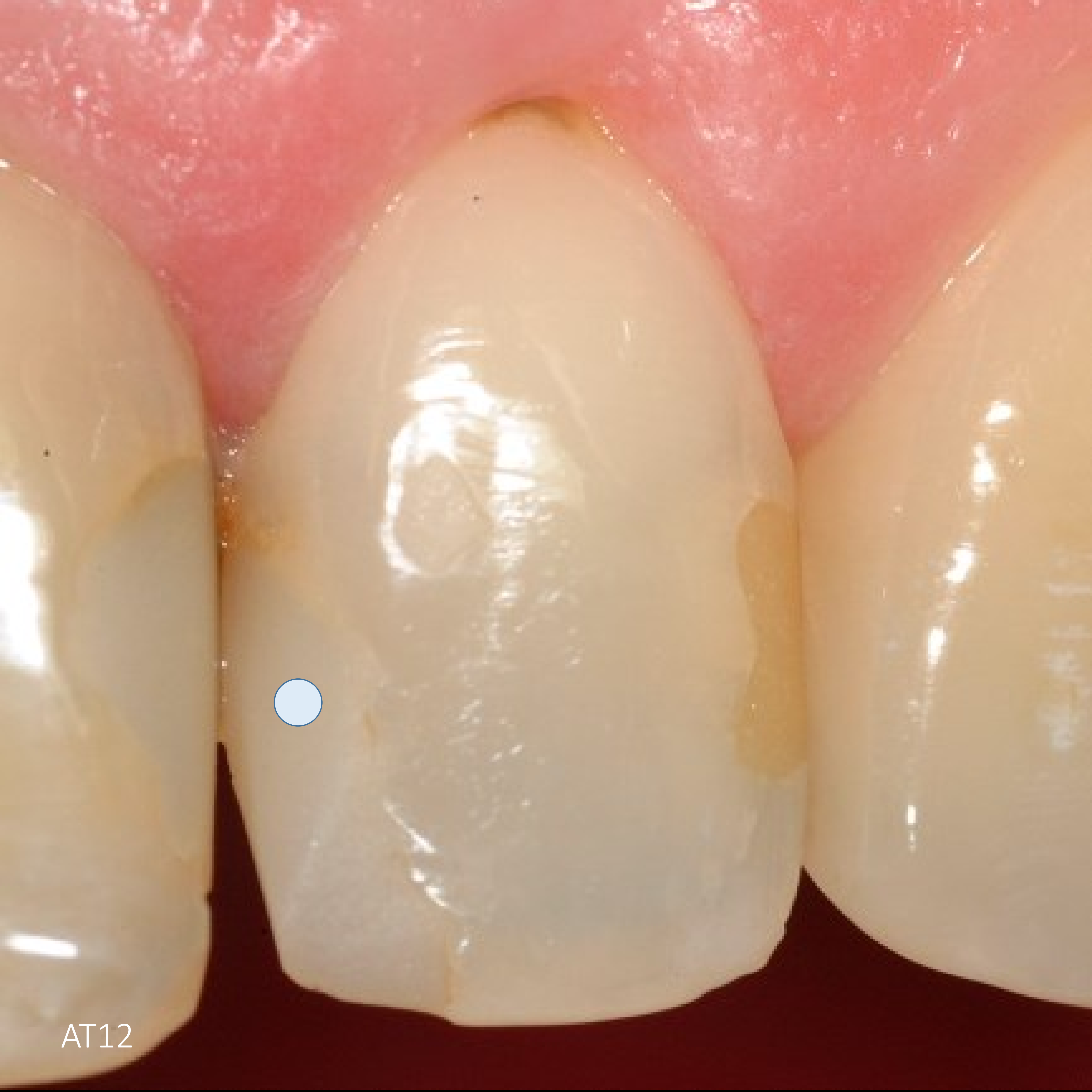
AT12

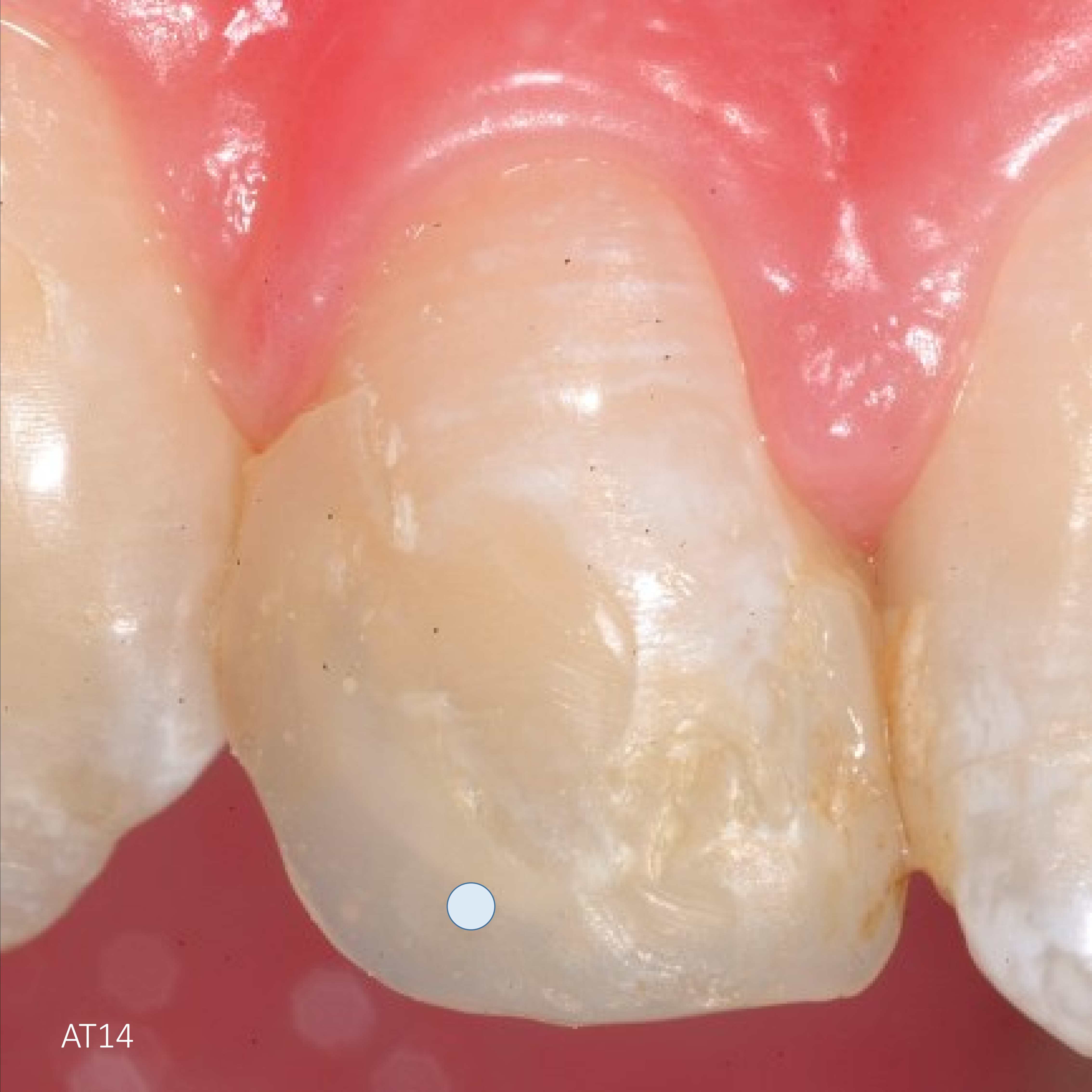
AT14

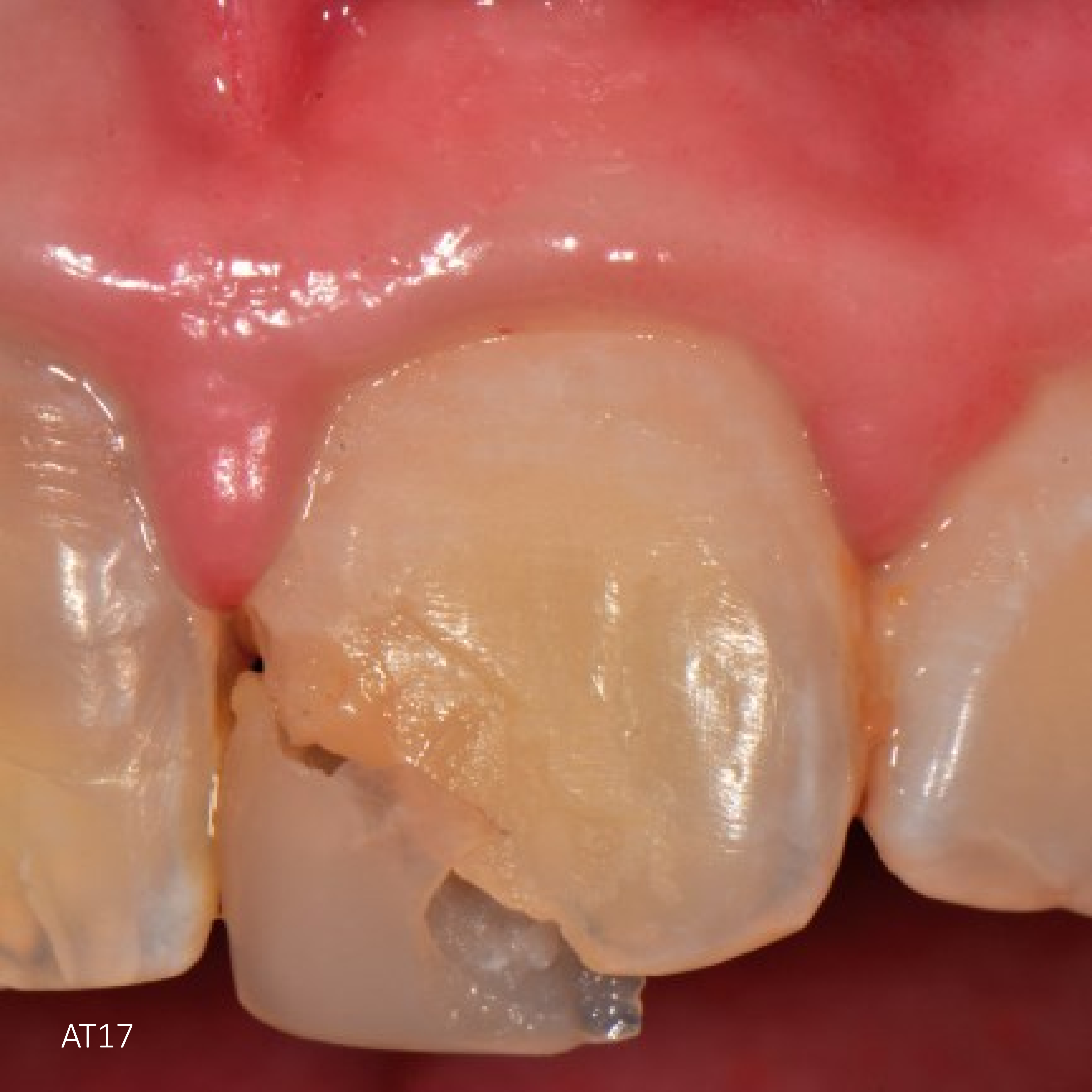
AT17

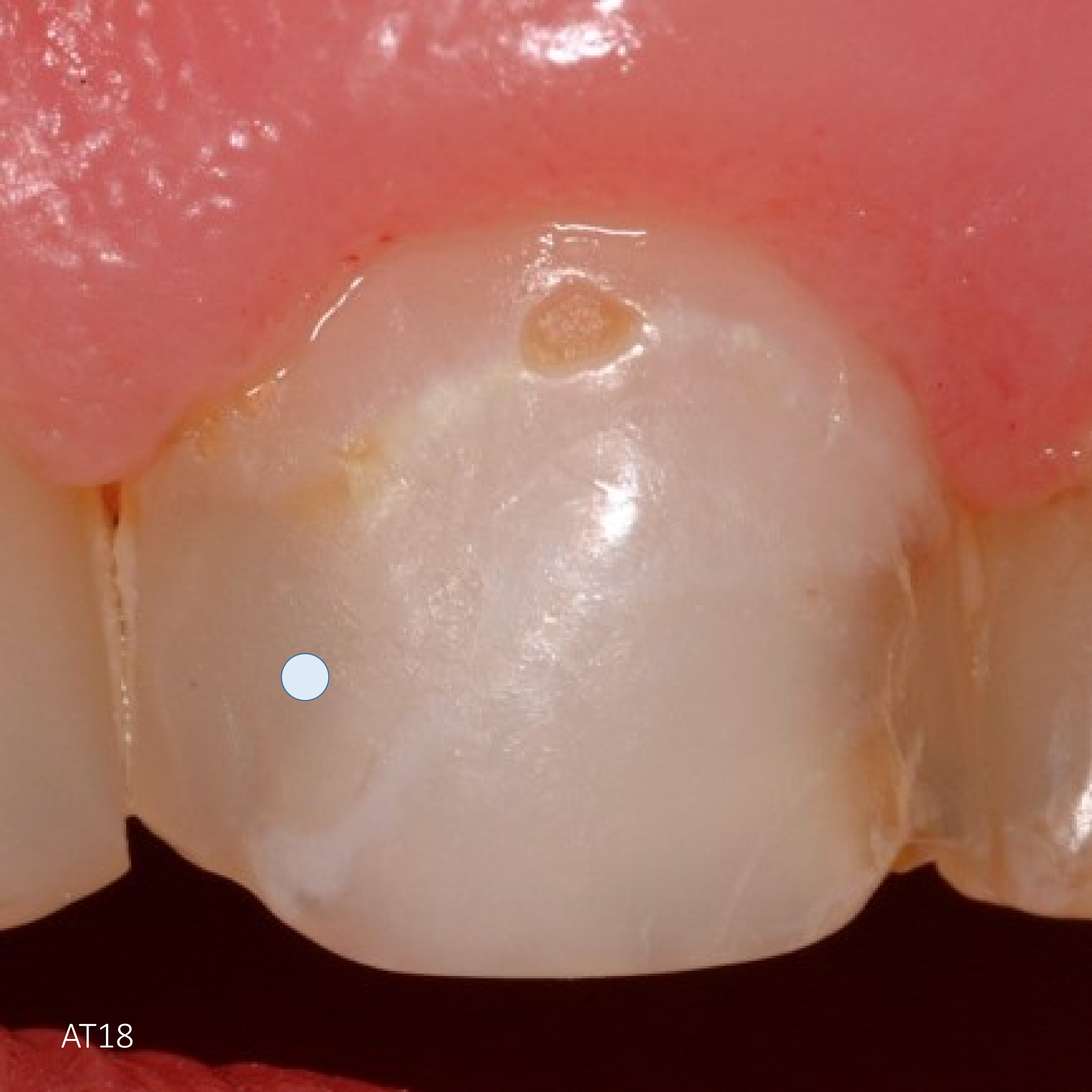
AT18

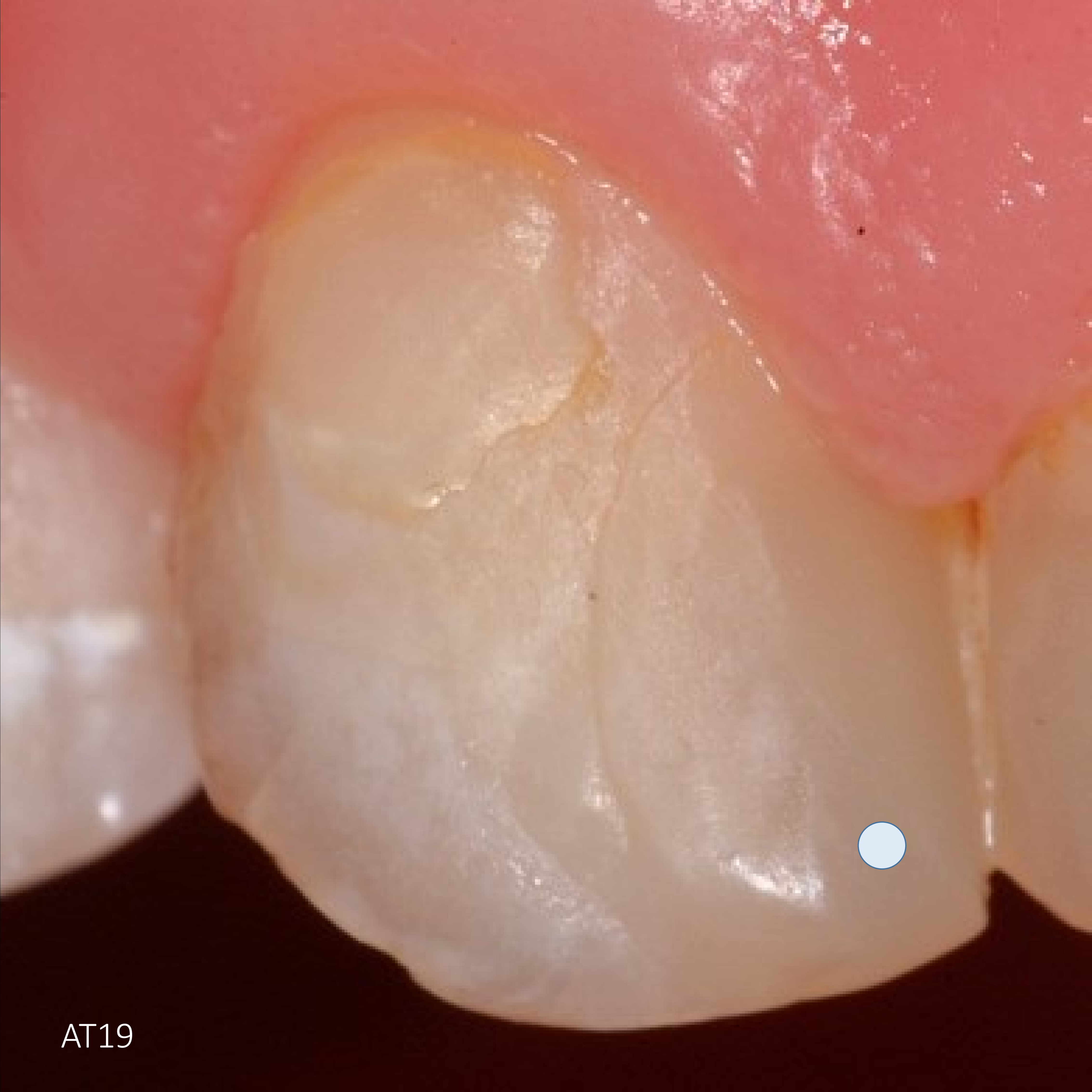
AT19

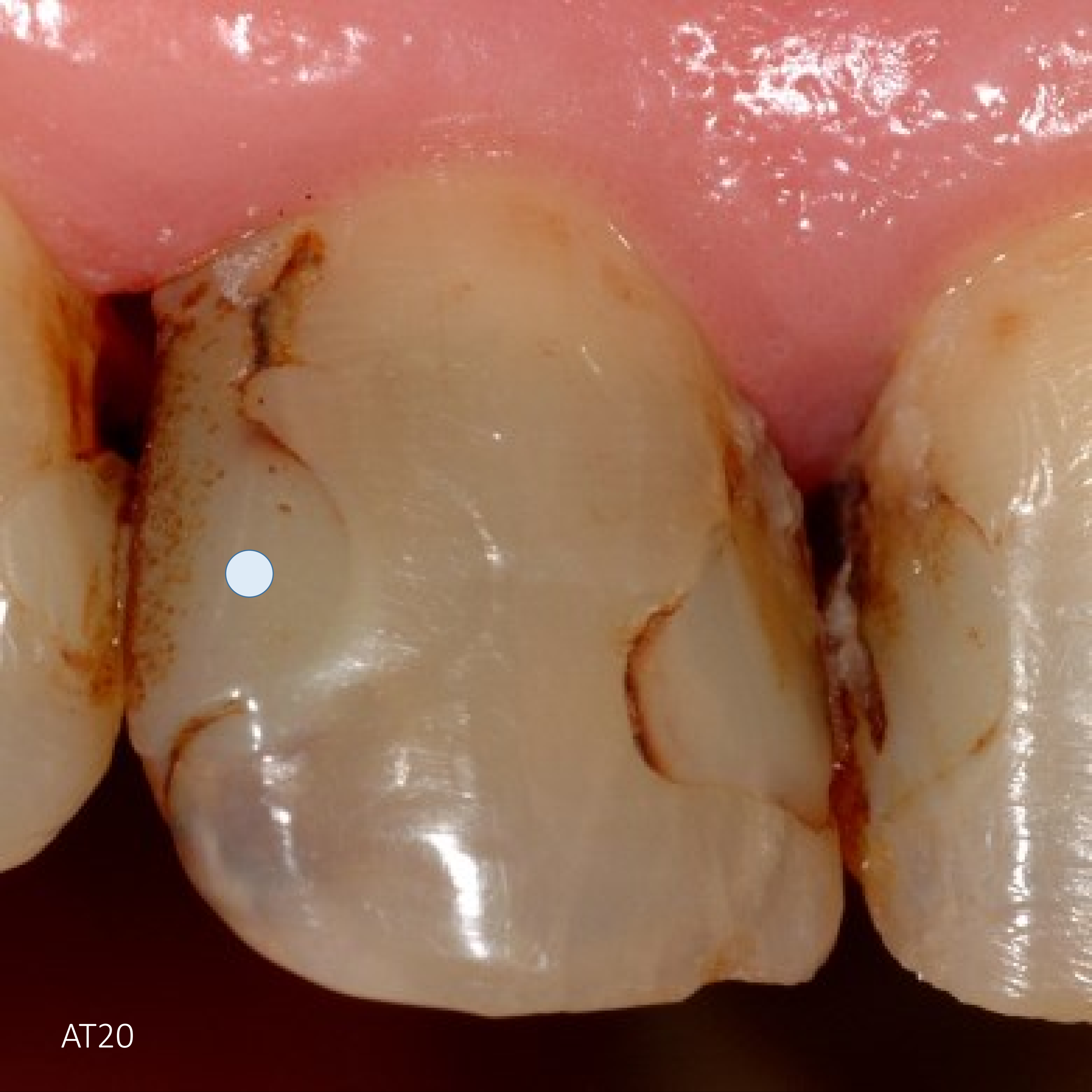
AT20

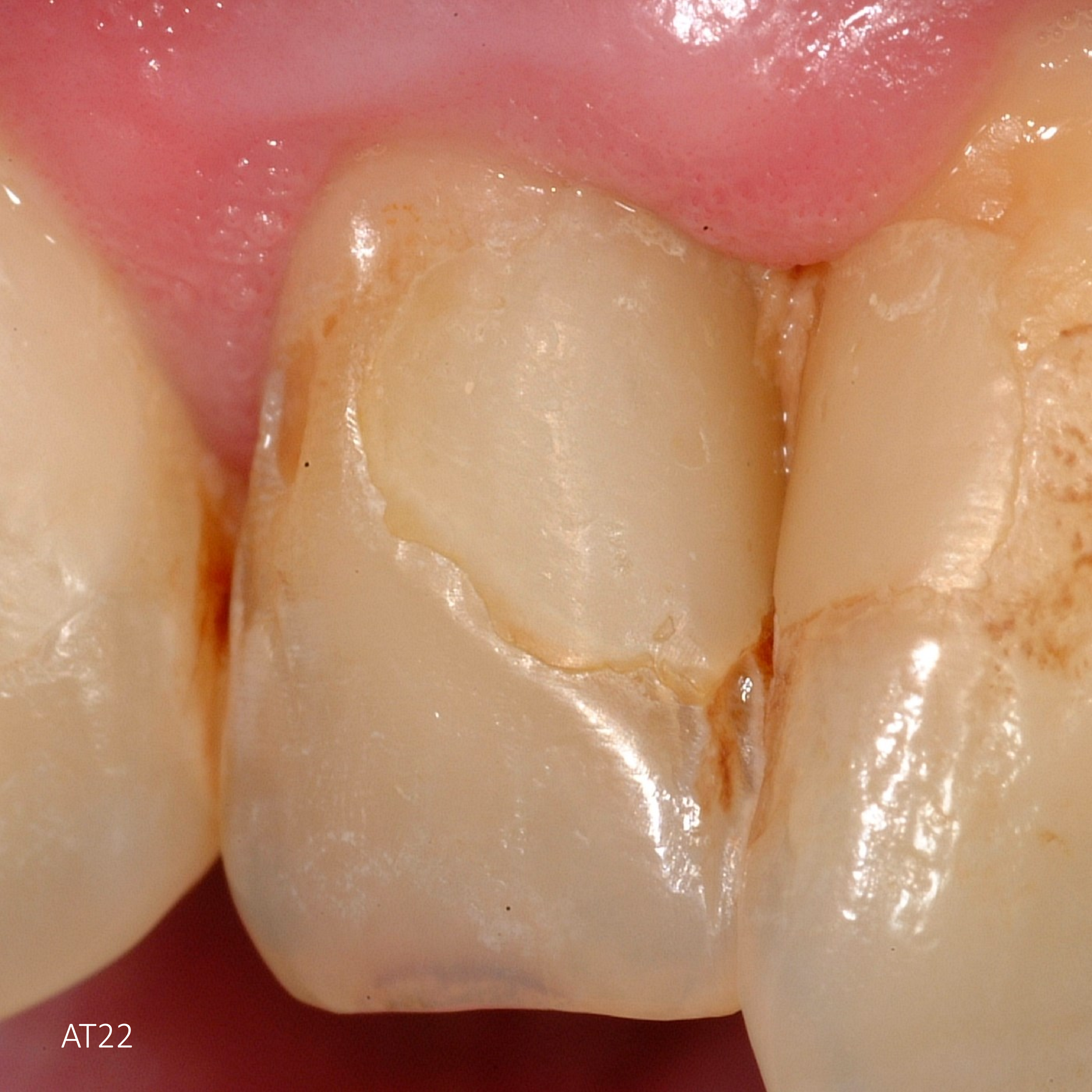
AT22

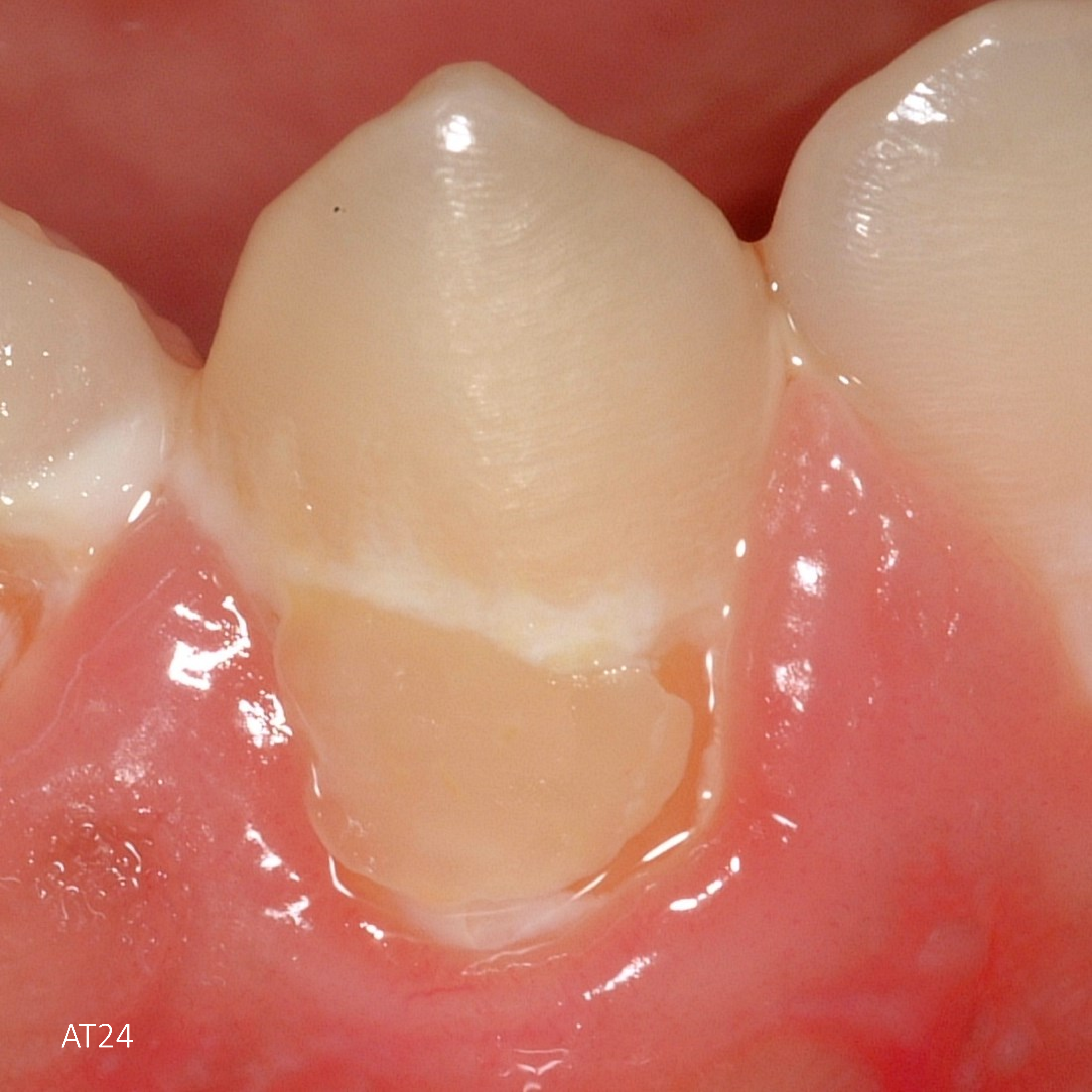
AT24

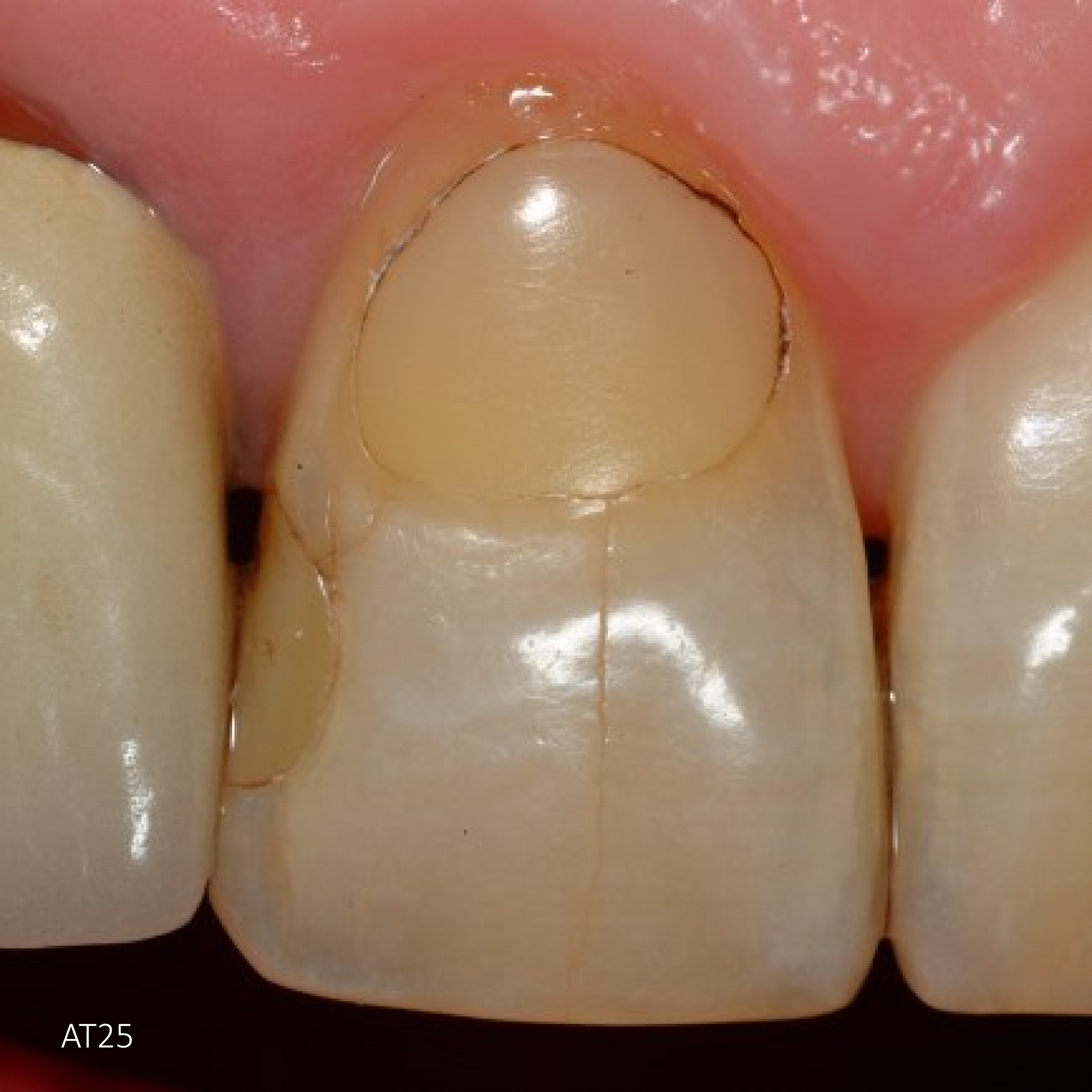

AT25

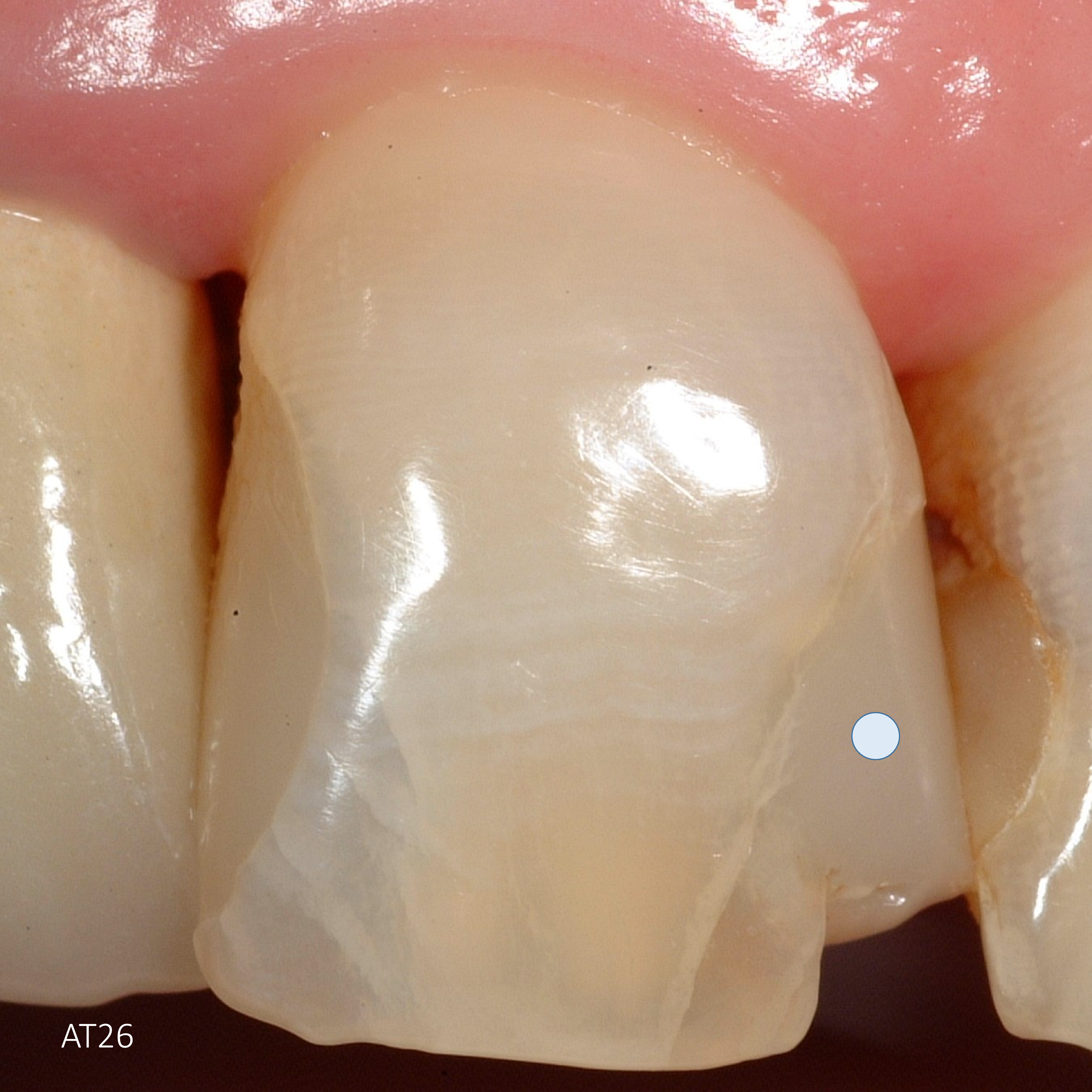
AT26

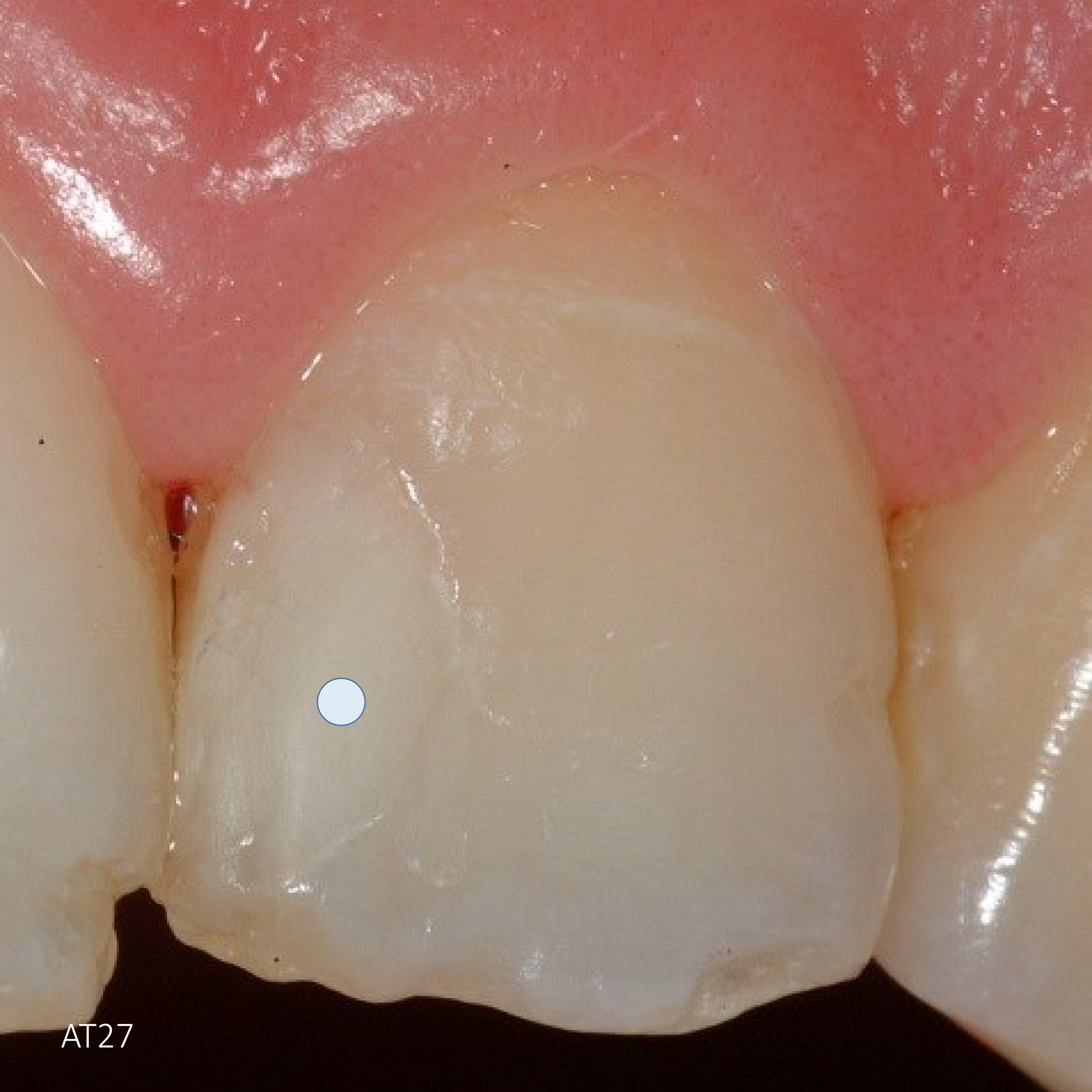
AT27

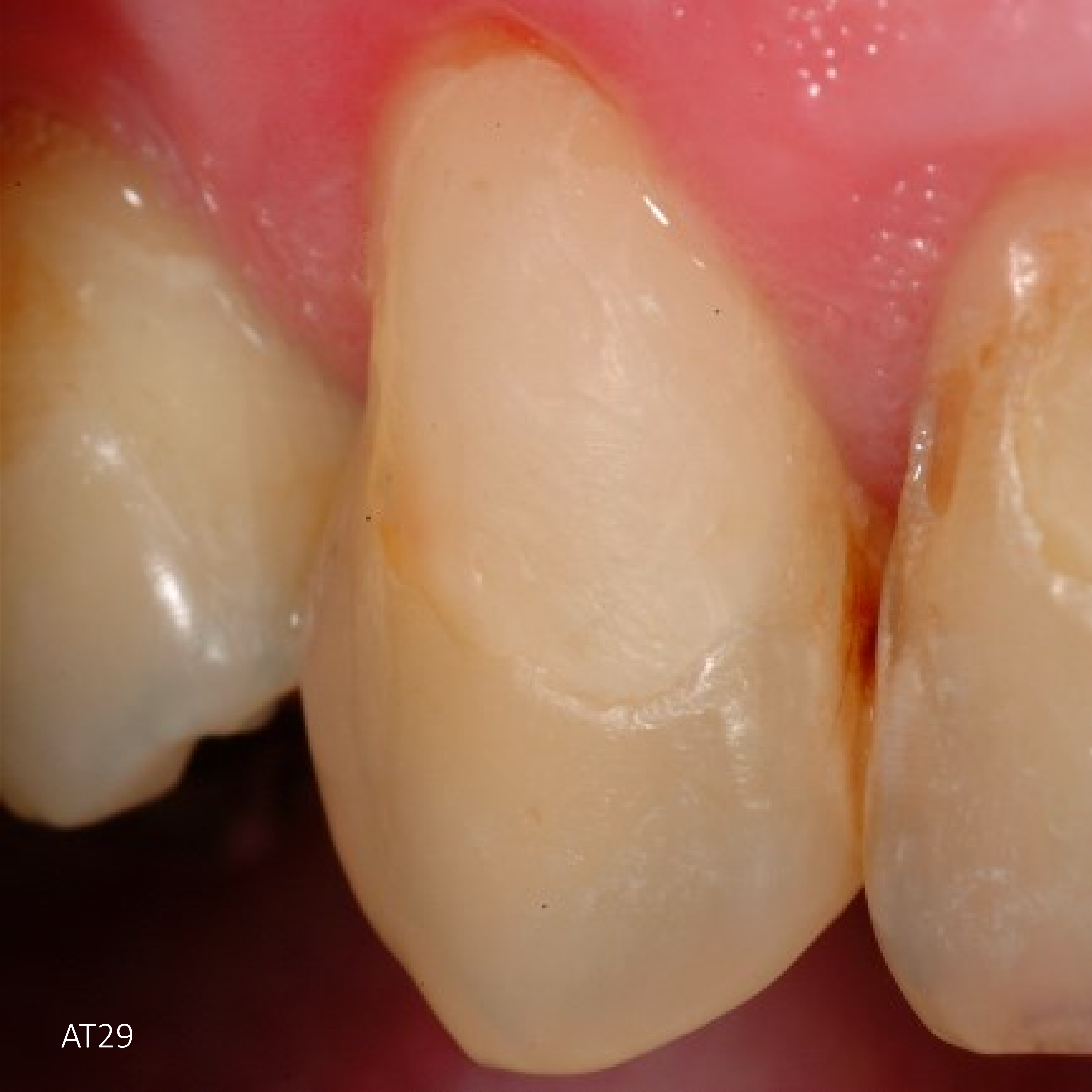
AT29

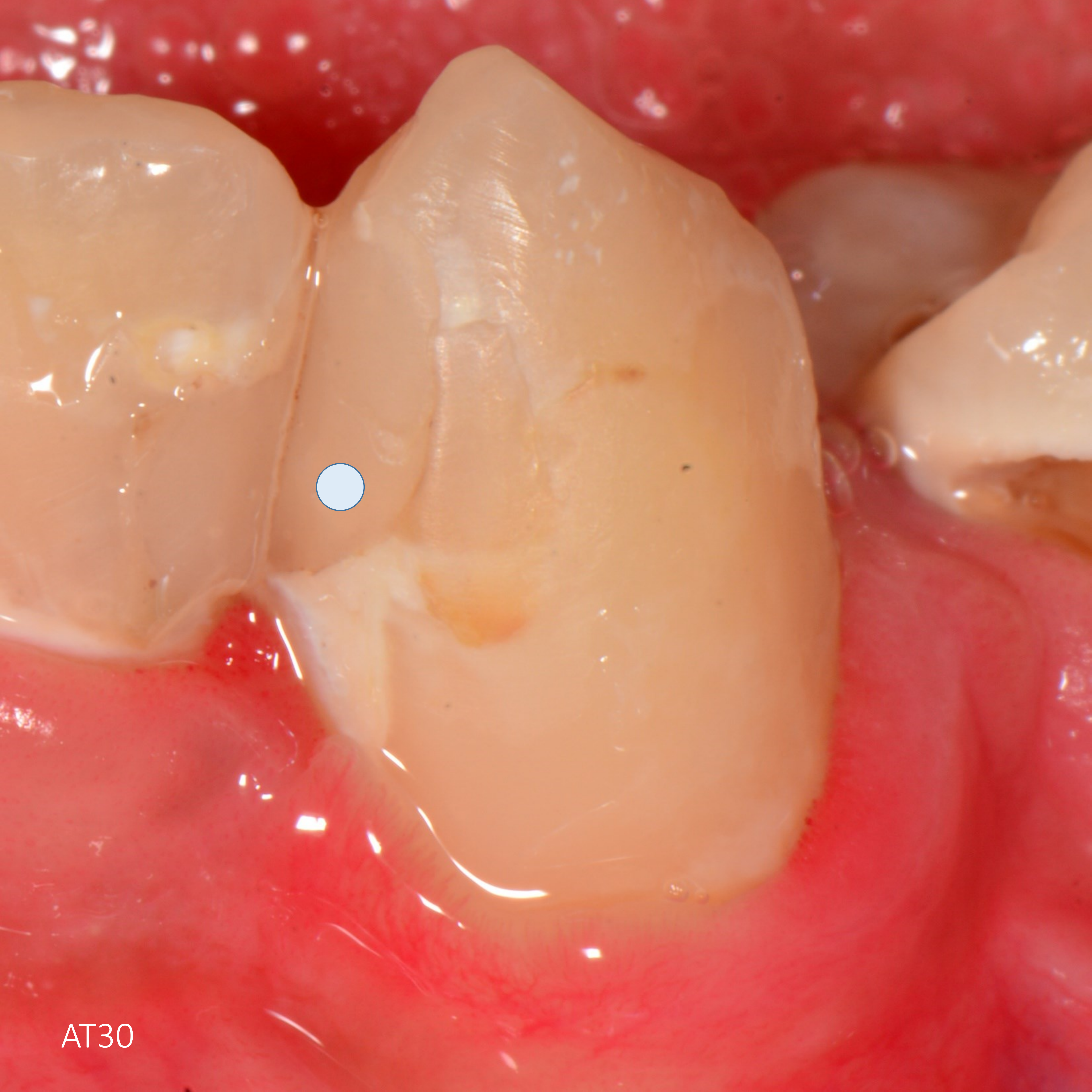
AT30

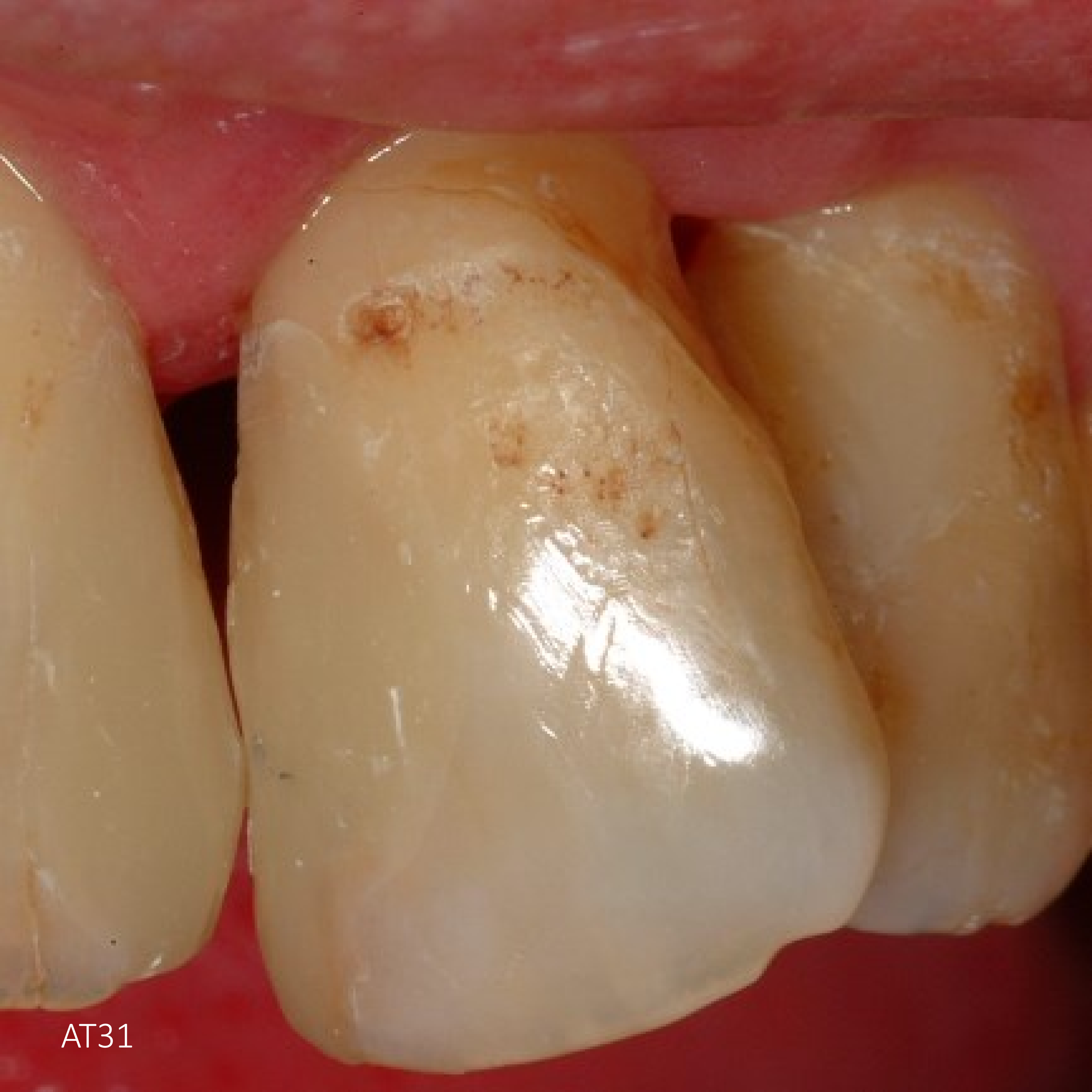
AT31

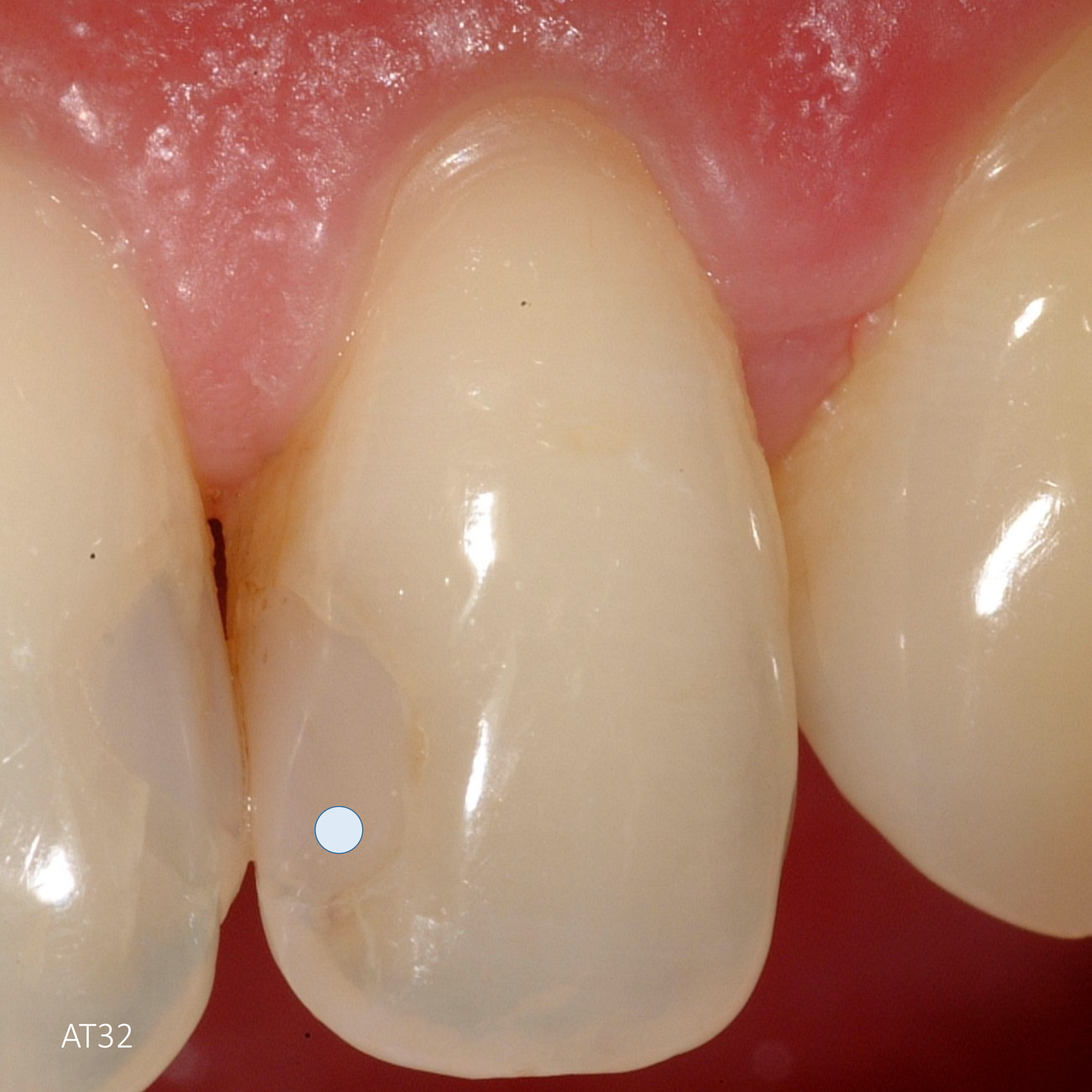
AT32

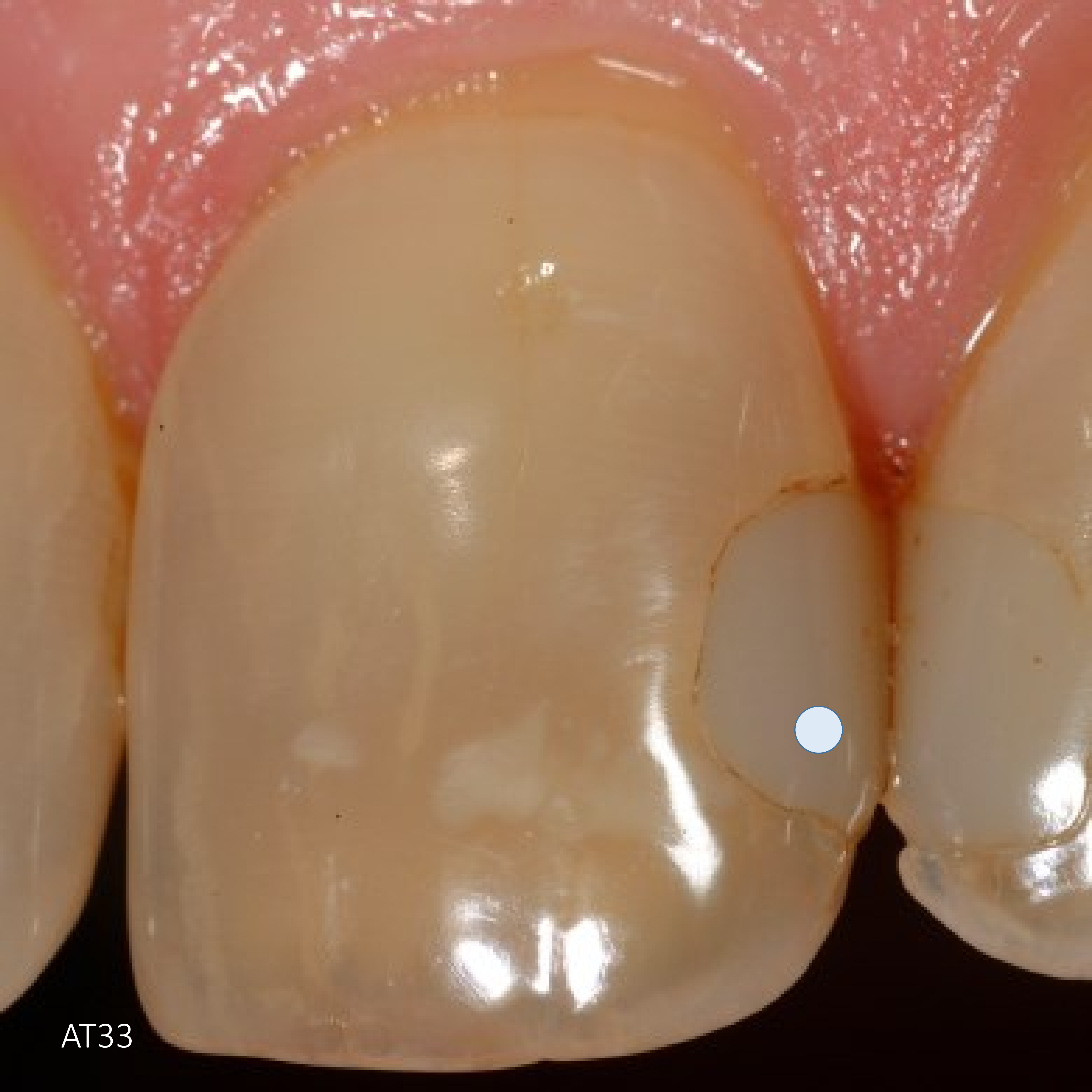
AT33

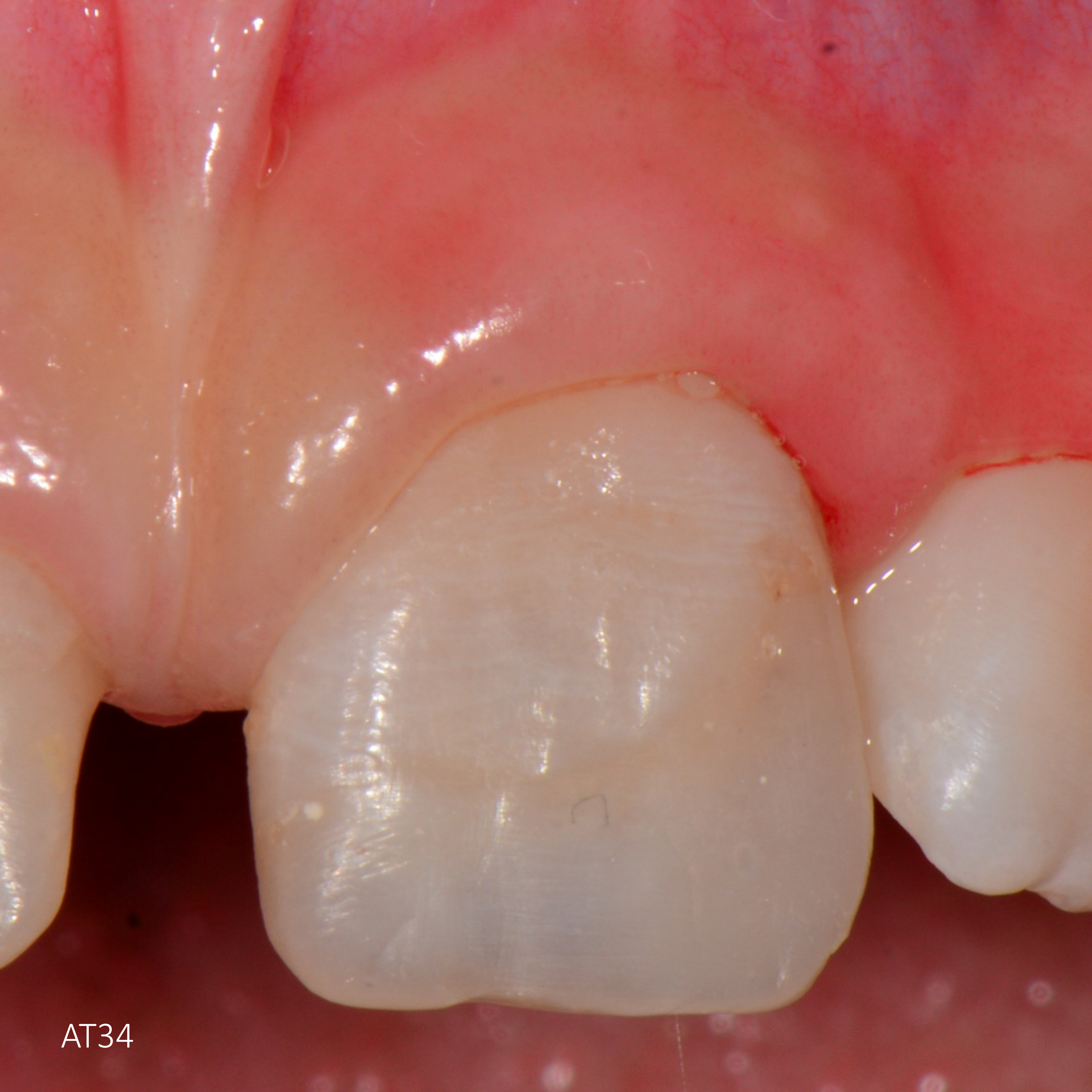

AT34

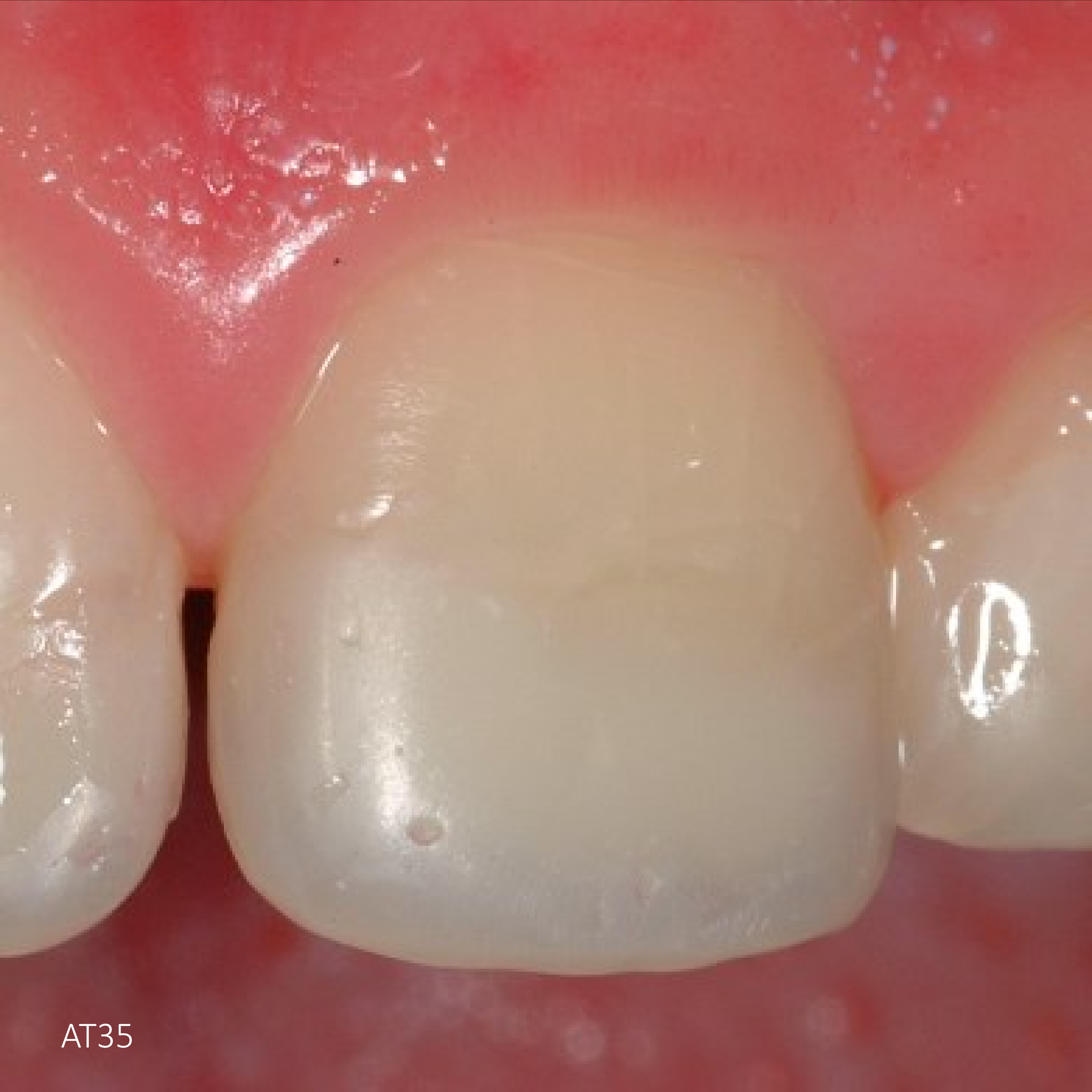
AT35

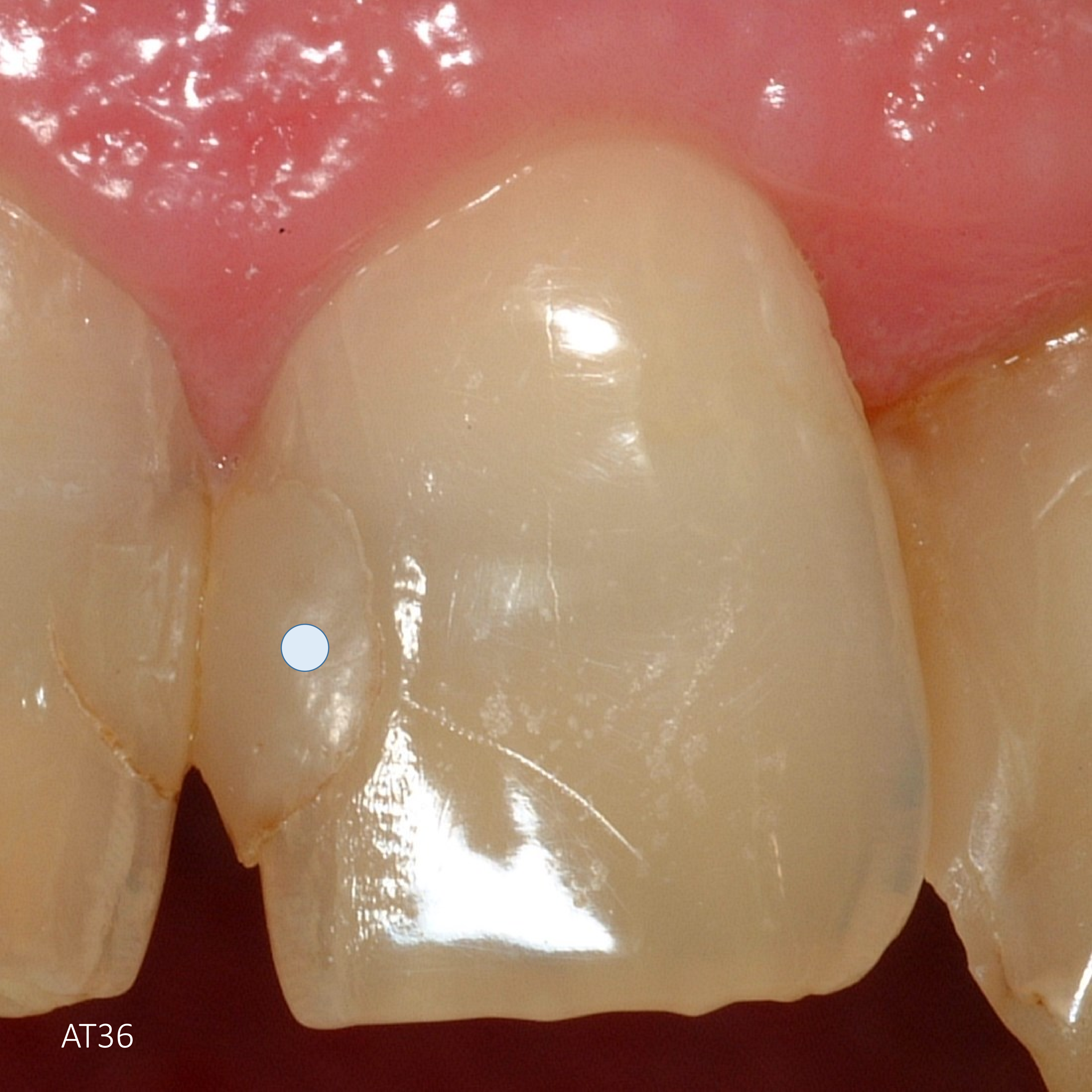
AT36

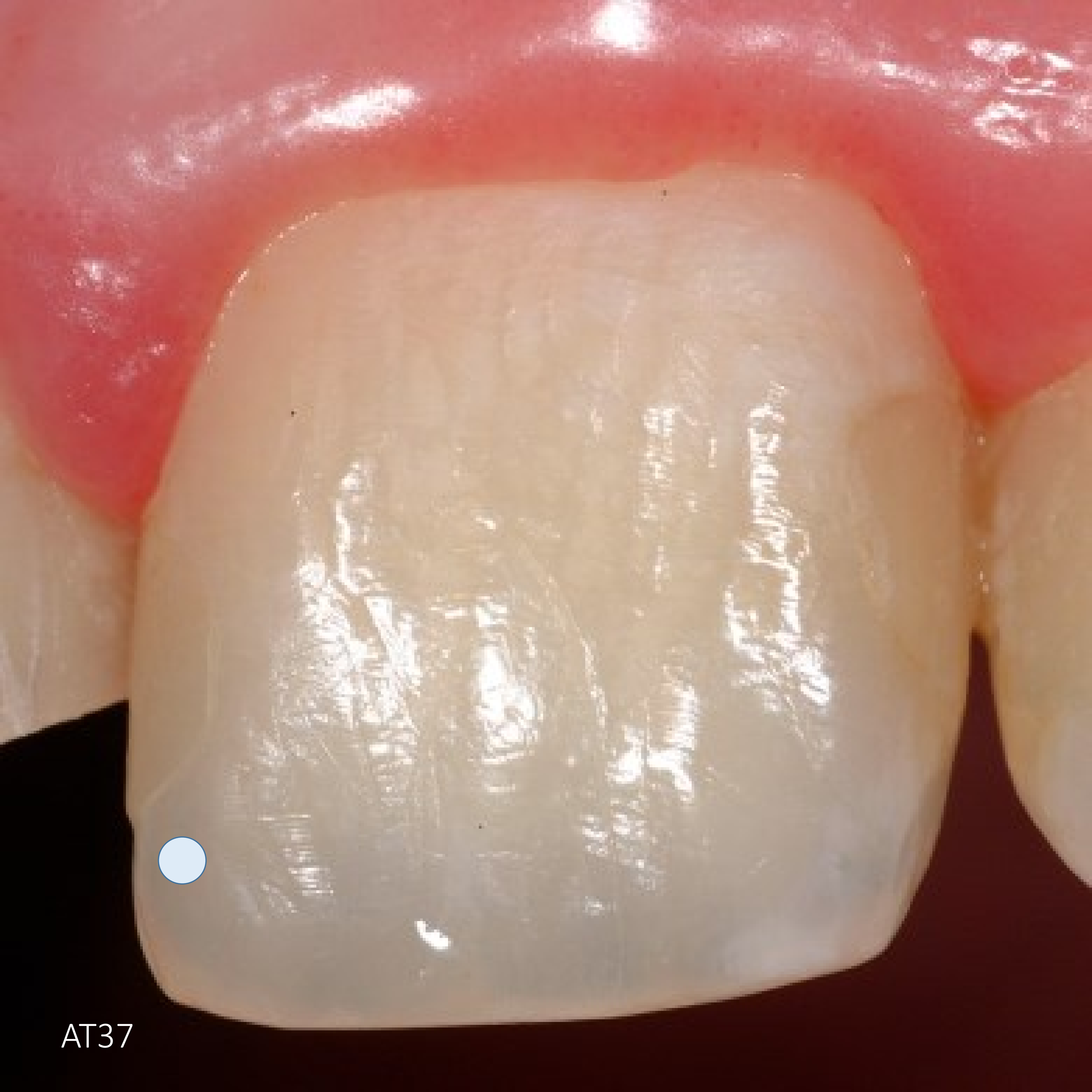
AT37

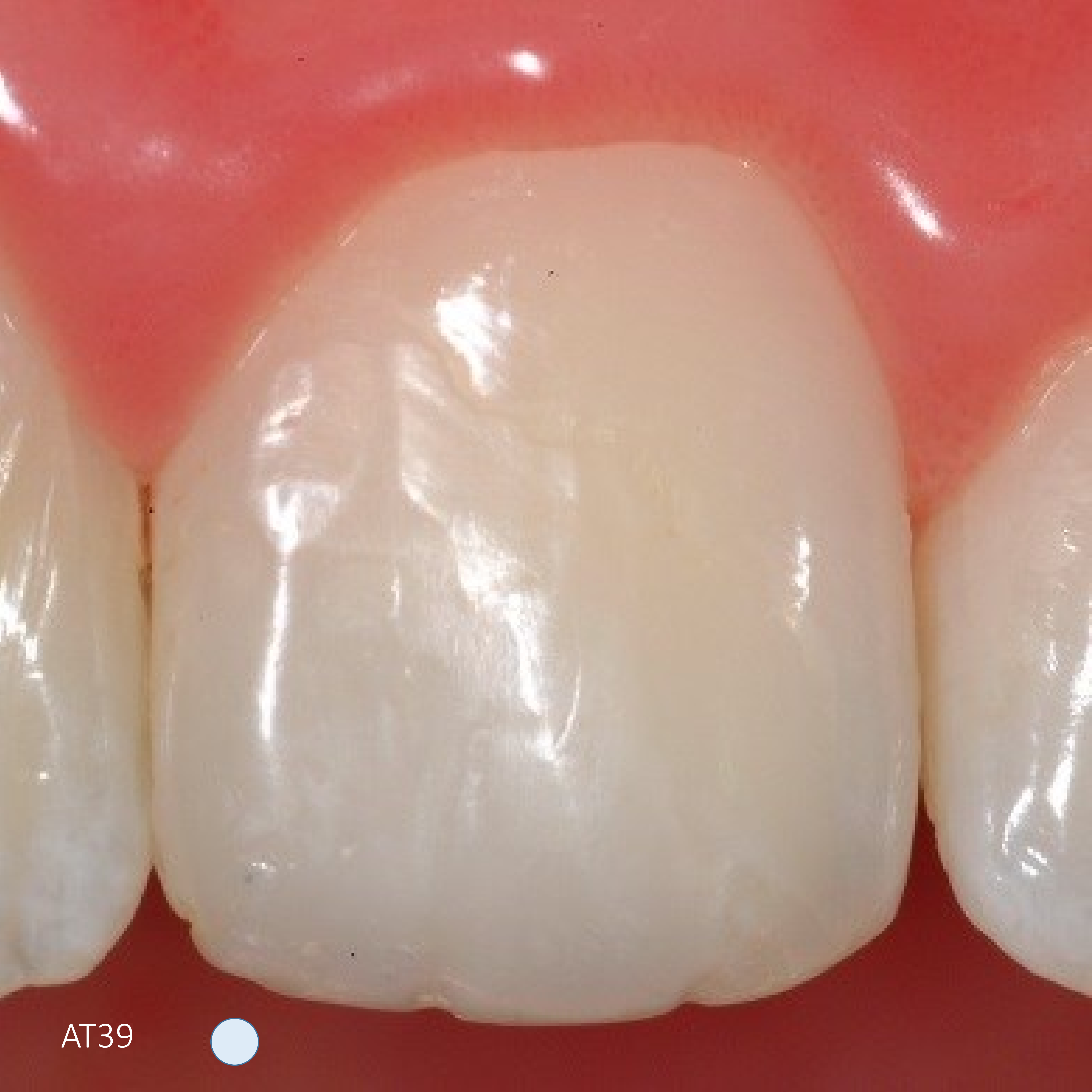
AT39
